# Body‐wide genetic deficiency of poly(ADP‐ribose) polymerase 14 sensitizes mice to colitis

**DOI:** 10.1096/fj.202400484R

**Published:** 2024-07-05

**Authors:** Madhukar Vedantham, Lauri Polari, Anbu Poosakkannu, Rita G. Pinto, Moona Sakari, Jukka Laine, Petra Sipilä, Jorma Määttä, Heidi Gerke, Tiia Rissanen, Pia Rantakari, Diana M. Toivola, Arto T. Pulliainen

**Affiliations:** ^1^ Institute of Biomedicine University of Turku Turku Finland; ^2^ Cell Biology, Biosciences, Faculty of Science and Engineering Åbo Akademi University Turku Finland; ^3^ InFLAMES Research Flagship Center Åbo Akademi University Turku Finland; ^4^ Turku Center for Disease Modeling University of Turku Turku Finland; ^5^ Department of Pathology Turku University Hospital Turku Finland; ^6^ Turku Bioscience Centre University of Turku and Åbo Akademi University Turku Finland; ^7^ InFLAMES Research Flagship Center University of Turku Turku Finland; ^8^ Department of Biostatistics University of Turku Turku Finland

**Keywords:** colitis, Crohn's disease, inflammation, inflammatory bowel disease, Parp14, ulcerative colitis

## Abstract

Inflammatory bowel disease (IBD) is a chronic disease of the gastrointestinal tract affecting millions of people. Here, we investigated the expression and functions of poly(ADP‐ribose) polymerase 14 (Parp14), an important regulatory protein in immune cells, with an IBD patient cohort as well as two mouse colitis models, that is, IBD‐mimicking oral dextran sulfate sodium (DSS) exposure and oral *Salmonella* infection. Parp14 was expressed in the human colon by cells in the lamina propria, but, in particular, by the epithelial cells with a granular staining pattern in the cytosol. The same expression pattern was evidenced in both mouse models. Parp14‐deficiency caused increased rectal bleeding as well as stronger epithelial erosion, Goblet cell loss, and immune cell infiltration in DSS‐exposed mice. The absence of Parp14 did not affect the mouse colon bacterial microbiota. Also, the colon leukocyte populations of Parp14‐deficient mice were normal. In contrast, bulk tissue RNA‐Seq demonstrated that the colon transcriptomes of Parp14‐deficient mice were dominated by abnormalities in inflammation and infection responses both prior and after the DSS exposure. Overall, the data indicate that Parp14 has an important role in the maintenance of colon epithelial barrier integrity. The prognostic and predictive biomarker potential of Parp14 in IBD merits further investigation.

## Graphical abstract

Parp14 has a role in the maintenance of colon epithelial barrier integrity in a mouse model of inflammatory bowel disease. Parp14‐deficient mice displayed increased rectal bleeding as well as stronger epithelial erosion, Goblet cell loss, and immune cell infiltration in the distal colon upon colitis‐inducing oral dextran sulfate sodium (DSS) exposure. RNA‐Seq demonstrated that the colon transcriptomes of Parp14‐deficient mice were dominated by abnormalities in inflammation and infection responses both prior and after the DSS exposure.

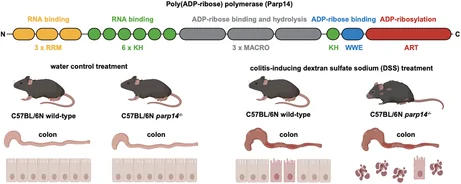

**Abbreviations**
ART: ADP‐ribosyltransferase
CD: Crohn's disease
DEG: differentially expressed gene
DSS: dextran sulfate sodium
FFPE: formalin‐fixed paraffin‐embedded
GO: gene ontology
H&E: hematoxylin and eosin
IBD: inflammatory bowel disease
IHC: immunohistochemistry
KEGG: Kyoto Encyclopedia of Genes and Genomes
OTU: operational taxonomy unit
PBS: phosphate buffered saline
Parp14: poly(ADP‐ribose) polymerase 14
UC: ulcerative colitis

## 1 INTRODUCTION

Inflammatory bowel disease [IBD, ulcerative colitis (UC), and Crohn's disease (CD)] is a debilitating and relapsing chronic disease of the gastrointestinal tract affecting millions of people, mostly diagnosed in adolescence or early adulthood.^1^ The etiology of IBD remains unknown, but it appears to involve a genetic predisposition. Approximately 200 risk loci have been identified with a major role in genes regulating immunological pathways controlling microbial recognition and killing, for example, in *Nod2* gene.^2^, ^3^ Indeed, infections with enteric pathogens such as *Salmonella* leading to gut microbiota dysbiosis and alterations of the intestinal immune responses have been debated as environmental factors.^2^ The disease culminates in dysfunction of the epithelial barrier, an increase in immune cell infiltration, and elevated concentrations of the inflammatory cytokines.^1^ IBD can be controlled in only a subset of patients, for example, with anti‐TNF‐α antibodies or leukocyte migration‐blocking anti‐α4β7 integrin antibodies,^3^ highlighting the need to identify new drug targets. Patients that do not respond develop adverse effects, most notably increased risk of infections and colorectal cancer, thus requiring continuous medical monitoring, and sometimes removal of the affected tissues by surgery is required.^1^, ^3^ There is a widespread consensus that a more individualized approach is required, with a need for more accurate prognostic and predictive biomarkers that allow more efficient patient stratification.

Poly(ADP‐ribose) polymerase 14 (Parp14) is an enzyme of the Parp protein family.^4^ Parp family members catalyze ADP‐ribosylation of macromolecules, that is, proteins and nucleic acids, which refers to the covalent conjugation of an ADP‐ribose moiety from nicotinamide adenine dinucleotide (NAD+) onto the substrate with simultaneous release of nicotinamide.^4^ Parp functions that are independent of the ADP‐ribosyltransferase (ART) activity, such as scaffolding and modification reversal, are also important, as exemplified by the recent work on ADP‐ribose binding and the ADP‐ribose hydrolysis activities of Parp14 macrodomains.^5^, ^6^ Parp14 was identified as a Stat6‐interacting protein,^7^ and accordingly acts as a transcriptional co‐factor in interleukin‐4 (IL‐4)‐induced Stat6‐dependent gene expression in B cells.^7^, ^8^, ^9^, ^10^ Parp14 has functions also in other lymphocytes, for example, T cells from Parp14‐deficient mice have difficulties to differentiate into the IL‐4/Stat6‐dependent Th2 direction.^11^, ^12^ Interestingly, Parp14 has negative downstream effects on interferon‐γ (IFN‐γ)‐ and Stat1‐dependent cellular responses. Most notably, genetic Parp14 inactivation in macrophages skews them toward a pro‐inflammatory IFN‐γ‐driven M1 phenotype while reducing the IL‐4‐driven M2 phenotype.^13^ Based on these opposing effects on IFN‐γ‐ and IL‐4‐mediated gene expression, Parp14 has been debated to play a role in macrophage polarization.^13^, ^14^, ^15^ ADP‐ribosylation of Stat proteins is one possible mechanism by which Parp14 may regulate macrophage functions.^13^, ^16^ Overall, Parp14 appears to be an important regulator of a number of different immune cell functions.

We hypothesized that Parp14 is involved in the colon immune responses, and, if so, its malfunction could play a role in the onset or development of IBD. We explored the effect of body‐wide genetic Parp14‐deficiency in the robust murine model of IBD, that is, in the 1‐week oral dextran sulfate sodium (DSS) exposure colitis model.^17^, ^18^ We also conducted a histological survey of Parp14 expression in the mouse colon in the 1‐week oral DSS exposure colitis model and, in parallel, in the single‐dose oral *Salmonella* exposure colitis model,^19^ and in the human colon using colon biopsies of IBD patients. The data highlight Parp14 as a protein highly expressed by epithelial cells and having an important role in the maintenance of colon epithelial barrier integrity.

## 2 MATERIALS AND METHODS

### 2.1 Expression and purification of the GST‐Parp14 fragment

#### 2.1.1 Plasmid

A synthetic DNA fragment (Eurofins Genomics) encoding for amino acids 291–358 of the human Parp14 (Uniprot Q460N5), that is, the epitope of the sc‐377150 antibody (Santa Cruz Biotechnology), was cloned with BamHI and XhoI into pGEX‐6‐P1 (N‐terminal GST‐tag, pGEX‐6‐P1‐PARP14^291–358^).

#### 2.1.2 Expression

The pGEX‐6‐P1‐PARP14^291–358^ plasmid was transformed into BL21(DE3) (Novagen) and selected overnight at 37°C on Luria‐Bertani agar with 200 μg/mL of ampicillin. The next day, one colony was inoculated to 5 mL of liquid Luria–Bertani with 200 μg/mL of ampicillin, and grown overnight at 37°C with shaking. In the subsequent day, the bacteria were subcultured to Luria–Bertani 1:100 with 200 μg/mL of ampicillin and grown at 37°C with shaking until the OD_600_ reached 0.5 upon which isopropyl β‐d‐1‐thiogalactopyranoside was added to 500 μM. The culture was grown at room temperature with shaking for 4 h. Bacteria were collected by centrifugation and frozen to −80°C.

#### 2.1.3 Purification

Lysozyme (Sigma, L6876) and protease inhibitors (Thermo Fisher Scientific, A32965) were added to 0.5 mg/mL and 1 tablet/50 mL, respectively, in the thawed biomass in lysis buffer [50 mM Tris (pH 8.0), 150 mM NaCl, 2 mM dithiotreitol (DTT)]. The sample was sonicated and clarified by centrifugation. The supernatant was incubated with Protino Glutathione Agarose 4B beads (Macherey‐Nagel, 745500) in a rotatory shaker overnight at +4°C. The beads were applied to an empty plastic chromatography column and washed with gravity flow using 50 mM Tris (pH 8.0), 150 mM NaCl, 2 mM DTT. The GST‐PARP14^291–358^ protein was eluted with 50 mM Tris (pH 8.0), 150 mM NaCl, 2 mM DTT, 25 mM glutathione (Sigma, G4251), aliquoted, and stored at −80°C.

### 2.2 Patient material and Parp14 immunohistochemistry

Formalin‐fixed paraffin‐embedded (FFPE) biopsy specimens sectioned at 5 μm thickness from anonymous IBD (UC and CD) versus control patients were obtained from the Finnish biobank system (https://site.fingenious.fi/en/, Auria Biobank, Turku, Finland, project number ab20‐3646). The FFPE sections were stained for Parp14 with mouse monoclonal antibody (sc377150, Santa Cruz Biotechnology, dilutions 1:500, 1:1000, and 1:10 000) and detected using the mouse‐specific HRP‐DAB (ABC) detection immunohistochemistry (IHC) kit (ab64259, Abcam). Tissue sections were air dried for 2 h at room temperature, placed in a 37°C incubator overnight, deparaffinized in xylene, and rehydrated with alcohol gradients. Endogenous peroxidase was blocked with the ‘Peroxidase blocking solution’ provided with the IHC kit. The sections were immersed in prewarmed 10 mM Na‐citrate buffer (pH 6.0) and kept in a boiling water bath for 20 min. After antigen retrieval, sections were rinsed in PBST [phosphate buffered saline (PBS) with 0.01% Tween‐20], followed by BSA blocking (5% w/v in PBST) for 1 h at room temperature to reduce nonspecific binding of the antibodies. The primary anti‐Parp14 antibody in BSA (5% w/v in PBST) was added to tissue sections and incubated overnight at 4°C in a humidified chamber. Post‐incubation with primary antibody, tissue sections were rinsed in PBST twice for 5 min each and incubated with biotinylated anti‐mouse secondary antibody (provided with the IHC kit) for 1 h at room temperature. Streptavidin‐HRP conjugate was added and then stained using 3–3′ Diaminobenzidine (DAB) as a chromogen (both solutions were provided with an IHC kit). Harris hematoxylin was used as a nuclear counterstain. Sections without incubation with the primary antibody served as negative controls. Mounting was done using Histo‐Clear, and sections were allowed to sit at room temperature for 12 h before imaging. Imaging was done using a Zeiss AxioImager M1 microscope with 5×, 20×, and 40× oil or 63× oil objective lenses. Two persons, one being a pathologist, independently and blind for the sample group, scored the stained sections (1:10 000 of anti‐Parp14 antibody) by visual inspection using a bright field microscope. The Parp14 staining intensity (0—none, 1—faint and irregular, 2—mild and regular, 3—strong and highly regular) was scored for the surface epithelial cells and the cryptal epithelial cells (see Figure 1C). The score of one particular patient means the average of scores from all the available tissue sections across the gastrointestinal tract, which varied from patient to patient. The scores of the two persons were averaged. Differences between cryptal and surface epithelial cell staining intensities in patient groups were summarized with descriptive statistics and studied by the Kruskal–Wallis test. The normality of variables was evaluated visually and tested with the Shapiro–Wilk test. Due to the non‐normality of the continuous variables, nonparametric methods were used. The statistical significance level was set at .05 in all tests (two‐tailed). The analyses were performed using the SAS system, version 9.4 for Windows (SAS Institute Inc., Cary, NC, USA).

**FIGURE 1:**
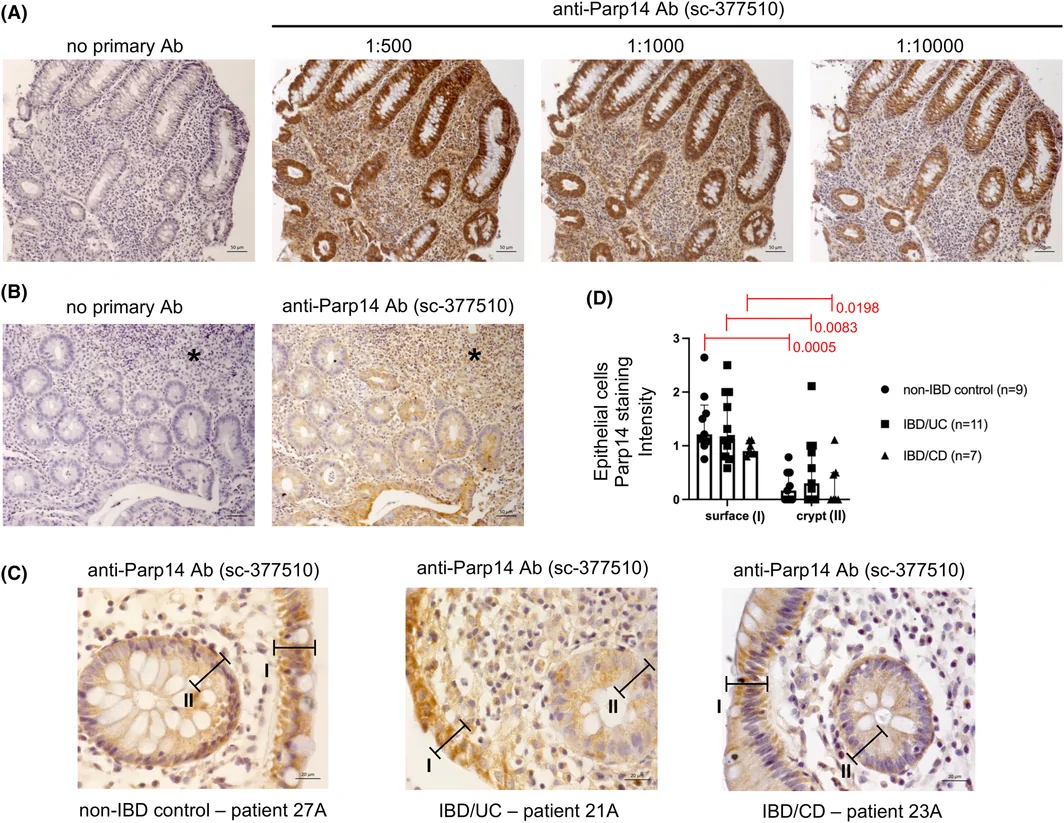
Parp14 expression and localization in the human colon. (A) IHC staining of Parp14 in an FFPE endoscopic biopsy section of an UC patient (TCML_VM_07A, descending colon, File S1). 20× objective images are shown. (B) IHC staining of Parp14 in an FFPE endoscopic biopsy section of an UC patient (TCML_VM_21A, ascending colon, File S1). 20× objective images are shown (1:10 000 dilution of anti‐Parp14 antibody). The star refers to a highly immune cell infiltrated site of the colon. (C) Examples of IHC staining of Parp14 in FFPE endoscopic biopsy sections of different patient groups (File S1). 63× objective images are shown (1:10 000 dilution of anti‐Parp14 antibody). The bars I and II refer to the areas that were visually scored by two independent persons for Parp14 staining intensity (D), that is, I—surface epithelial cells facing the lumen of the colon, and II—cryptal epithelial cells. (D) Statistical comparison of Parp14 staining intensity in different patient groups with the Kruskal–Wallis test. The analysis is based on staining intensity in surface and cryptal epithelial cells that were scored as 0, faint or negligible staining to 3, high intensity (10× objective images, 1:10 000 dilution of anti‐Parp14 antibody).

### 2.3 Validation of the anti‐Parp14 antibody

#### 2.3.1 Western analysis

A synthetic DNA fragment (Eurofins Genomics) encoding for the entire mouse Parp14 (Uniprot Q2EMV9) was cloned into pcDNA3.1‐Hygro(‐)‐based human EGFR‐HA expression plasmid.^20^ The human EGFR insert was replaced with the mouse Parp14 insert before the HA‐tag‐encoding area using NheI and NotI (C‐terminal HA‐tag, pcDNA3.1‐Hygro(‐)‐mParp14). The pcDNA3.1‐Flag‐Parp14 plasmid to express human Parp14 (N‐terminal Flag‐tag) has previously been described.^21^ HEK293T cells grown in DMEM (21969035, Thermo Fisher) + 10% FBS (S181B‐500, Biowest) + 2 mM l‐glutamine (25030081, Gibco) + 25 mM HEPES (15630‐056, Gibco) were seeded in 10 mL volumes in 10 cm dishes (1 × 10^6^ cells/10 cm dish) in the late afternoon. The next morning, fresh media was exchanged, and the cells were transfected with 4 μg of plasmid DNA using calcium‐phosphate transfection reagents (prepared in‐house). At 24 h post‐transfection, cells were washed with ice‐cold sterile PBS and lysed in modified RIPA buffer (50 mM Tris pH 7.5, 400 mM NaCl, 1% NP‐40, 0.5% sodium deoxycholate, 0.1% sodium dodecyl sulfate, 1 mM EDTA), 75 μM Tannic acid (PARG inhibitor, 403040, Sigma Aldrich), 40 μM PJ‐34 (PARP inhibitor, J64413, Fischer Scientific), Pierce protease‐phosphatase inhibitor (A32961, Thermo Fisher Scientific) and cleared by high‐speed centrifugation at 4°C. Protein concentration was measured from the supernatants with a Bradford protein assay. The heated (10 min, 95°C) samples in Laemmli loading dye were run on SDS‐PAGE (30 μg/lane) and transferred to a nitrocellulose membrane, followed by blocking with 5% (w/v) skimmed milk in Tris‐buffered saline with 0.1% Tween 20 detergent (TBST). A primary antibody solution was prepared with 5% (w/v) skimmed milk in TBST with anti‐Parp14 mouse monoclonal antibody (1:500, sc377150, Santa Cruz Biotechnology) and enough volume for two membranes (epitope competition vs. control). With the epitope competition, one‐half of this primary antibody solution was incubated in a rotary platform for 30 min at room temperature with GST‐Parp14^291–358^ protein (concentration 15× more than with the primary antibody) prior to incubation with the membrane in a rotary platform for 48 h at 4°C. Membranes were washed thrice with TBST containing 5% (w/v) skimmed milk for 10 min each time. Membranes were incubated in TBST containing 5% (w/v) skimmed milk with goat anti‐mouse IgG conjugated to horseradish peroxidase (HRP) (1:2000, 1010‐05, SouthernBiotech) for 3 h at 4°C in a rotary platform and washed thrice with TBST for 10 min each time. Membranes were subsequently developed with WesternBrightECL (Advansta) and imaged on ImageQuant LAS 4000 (GE Healthcare). Post‐imaging, the same membranes were probed with anti‐beta actin HRP conjugate (sc‐47778 HRP, Santa Cruz Biotechnology). Membranes were developed and imaged as above.

#### 2.3.2 Immunohistochemistry

FFPE sections of an UC patient (TCML_VM_28A, ascending colon, see File S1) were prepared for IHC as described above. The primary anti‐Parp14 antibody in BSA (5% w/v in PBST) was added to tissue sections and incubated overnight at 4°C in a humidified chamber. The epitope competition slide was processed in parallel in a similar manner, but the primary anti‐Parp14 antibody solution was pre‐incubated for 30 min at 4°C using 15× more GST‐Parp14^291–358^ protein than the primary antibody. Secondary antibody treatment and counterstaining were the same as described above. Imaging was done using a Zeiss AxioImager M1 microscope.

### 2.4 Western analysis of Parp14 expression in cell cultures upon cytokine stimulation

HeLa229 human cervical adenocarcinoma cells (CCL‐2.1, ATCC) grown in DMEM (21969035, Thermo Fisher) + 10% FBS (S181B‐500, Biowest) + 2 mM l‐glutamine (25030081, Gibco) + 25 mM HEPES (15630‐056, Gibco) were seeded in 3 mL volumes in 6‐well plates (3 × 10^5^ cells/well). THP‐1 human monocyte/macrophage cells (TIB20, ATCC) grown in RPMI 1640 (Lonza) + 10% FBS (S181B‐500, Biowest) were differentiated using 10 ng/mL PMA (phorbol 12‐myristate 13‐acetate, P8139, Sigma) for 24 h. THP‐1 cells were washed twice with sterile PBS, and fresh RPMI media without PMA was used to seed cells (2 × 10^5^ cells/well) in 3 mL volumes in 6‐well plates. Next morning, cells (HeLa229 and THP‐1) were washed again with PBS and exchanged with fresh media supplemented with 100 ng/mL of *E. coli* 0111: B4 strain derived LPS (tlrl‐3pelps, InvivoGen), 100 U/mL of IFN‐γ (PHC4031, Thermo Fisher Scientific), 100 U/mL of IFN‐α (SRP4596, Sigma Aldrich), or 10 ng/mL of TNFα (210‐TA, R&D systems). Wells with fresh media without any inflammatory stimuli served as controls. At times 2, 8, 12, 24 and 48 h post‐stimulation, cells were washed twice with sterile PBS, trypsinized, and lysed using ice‐cold NP‐40 lysis buffer (50 mM Tris pH 7.5, 1% NP‐40, 1× Pierce protease‐phosphatase inhibitors with EDTA). Sample preparation for SDS‐PAGE and Western analysis was done as described above. After blocking with TBST containing 5% (w/v) skimmed milk, membranes were probed with anti‐Parp14 mouse monoclonal antibody (1:500, sc‐377150, Santa Cruz Biotechnology) in a rotary platform for 48 h at 4°C. Membranes were washed thrice with TBST containing 5% (w/v) skimmed milk for 10 min each time. Membranes were incubated in TBST containing 5% (w/v) skimmed milk with mouse IgG kappa binding protein (m‐IgGκ BP) conjugated to HRP (1:2000) (sc‐516102, Santa Cruz Biotechnology) for 3 h at 4°C on a rotary and washed thrice with TBST for 10 min each time. Membranes were developed and imaged as described above. Post‐imaging, the same membranes were probed with anti‐beta actin HRP conjugated (sc‐47778 HRP, Santa Cruz Biotechnology) as a loading control. Membranes were developed and imaged as above.

### 2.5 Mouse experiments

The National Animal Experiment Board has approved our mouse experiments in the C57BL/6N background (Figure S4, salmonellosis—ESAVI/24418/2018, IBD—ESAVI/16359/2019). The samples of the previously reported DSS experiment in FVB/n background^22^ were re‐analyzed in this study.

#### 2.5.1 Colony breeding

We used the previously described body‐wide Parp14 knockout mice,^23^ kindly provided by Adam Hurlstone (University of Manchester, UK), in a specific‐pathogen‐free area at the Central Animal Laboratory of the University of Turku with free access to a soy‐free diet and water ad libitum. The Parp14 knockout mice were backcrossed for 10 generations to a C57BL/6N background before starting the experiments. Thereafter, heterozygous mice were inbred to generate homozygous littermates for experiments. The reported data is based on all the littermates that we were able to breed (see Figure 3A). Genotyping of mice was carried out from DNA extracted from earmarks of two‐week‐old to three‐week‐old mice by PCR (primer sequences for wt, 5‐GGCCTAACTATTCACTCGTGT‐3 (prAPV426) and 5‐CTGCTCTTCTAGATGATGCAGA‐3 (prAPV443); and primer sequences for Parp14 knockout mice, 5‐GATGCAACTGCAAGAGGGTTTAT‐3 (LTR rev_comp prAPV437) and 5‐CTGCTCTTCTAGATGATGCAGA‐3 (prAPV443)).

#### 2.5.2 DSS exposure

Male mice of age 6–8 weeks were used for the experiment. They were allocated to two treatment groups, that is, the control and DSS groups, with similar starting body weights (see Figure 3A). DSS (40 000 Da, TdB Consultancy AB, Uppsala, Sweden, #DB001) solution was fresh, dissolved in autoclaved water to a 2.5% (w/v) concentration, and administered to mice in drinking water during days 0–7 of the experiment.^17^, ^18^ Control mice were matched to DSS‐treated mice according to age and starting weight and were treated equally to DSS‐treated mice, except that they did not receive DSS. The mice were weighed daily during the DSS treatment. Stool consistency was also examined, and the presence of blood in the stool was measured during and after the DSS challenge. The mice were scored for presence of blood in stool (0 = none; 1 = small amounts of blood in stool pellets; 2 = blood found throughout pellet; 3 = clotted blood at anus; 4 = fresh blood on mice or on bedding materials of the cage), and stool consistency (1 = normal; 2 = formed but soft; 3 = slightly loose; 4 = liquid). Following treatment (see Figure 3A), mice were sacrificed by CO_2_ asphyxiation. Blood was collected by cardiac puncture. The colon was excised, its length measured, and then it was washed with PBS and cut into proximal, mid, and distal halves, which were studied separately. Spleens were also collected. Samples for RNA analysis (liquid N_2_) and histology (4% paraformaldehyde) were collected after colon tissues were cut in half longitudinally. Three variables (body weight, stool consistency, and blood in stool) were measured and summarized with descriptive statistics. For analyses, weight was converted to percentages. Differences between the groups were studied by the Friedman test, and differences in days were studied by the Wilcoxon signed rank test for each of the dependent variables (body weight, stool consistency, and blood in stool). The normality of variables was evaluated visually and tested with the Shapiro–Wilk test. Due to the non‐normality of the continuous variables, nonparametric methods were used. The statistical significance level was set at .05 in all tests (two‐tailed). The analyses were performed using the SAS system, version 9.4 for Windows (SAS Institute Inc., Cary, NC, USA).

#### 2.5.3 *Salmonella* infection

*Salmonella* mice experiments were performed according to Barthel and co‐workers.^19^ The naturally streptomycin‐resistant strain *S. enterica* serovar Tyhphimurium SL1344 was purchased from the Culture Collection University of Gothenburg (CCUG 51871), Gothenburg, Sweden. *Salmonella* bacteria were grown for 12 h at 37°C in Luria–Bertani with shaking, diluted 1:20 in fresh medium, and sub‐cultured for 4 h with shaking. Bacteria were washed twice and suspended in ice‐cold, sterile PBS. Female C57BL/6N mice of age 6–8 weeks were used for *Salmonella* infection experiments. They were allocated to two treatment groups, that is, control and *Salmonella* groups, with similar starting body weights (see Figure S4). Water and food were withdrawn 4 h before per os (p.o.) treatment with 20 mg of streptomycin (75 μL of sterile solution or 75 μL of sterile water [control]). Afterward, animals were supplied with water and food ad libitum. At 20 h after streptomycin treatment, water and food were withdrawn again for 4 h before the mice were infected with 10^8^ cfu of *Salmonella* (50 μL suspension in PBS p.o.) or treated with sterile PBS (control). Thereafter, drinking water ad libitum was offered immediately and food 2 h post‐infection (p.i.). At the indicated times p.i., mice were sacrificed by CO_2_ asphyxiation, and tissue samples from the intestinal tracts were removed for Parp14 immunohistochemical staining (sample collected in 4% paraformaldehyde) and RNA analysis (sample collected in liquid N_2_).

**FIGURE 2:**
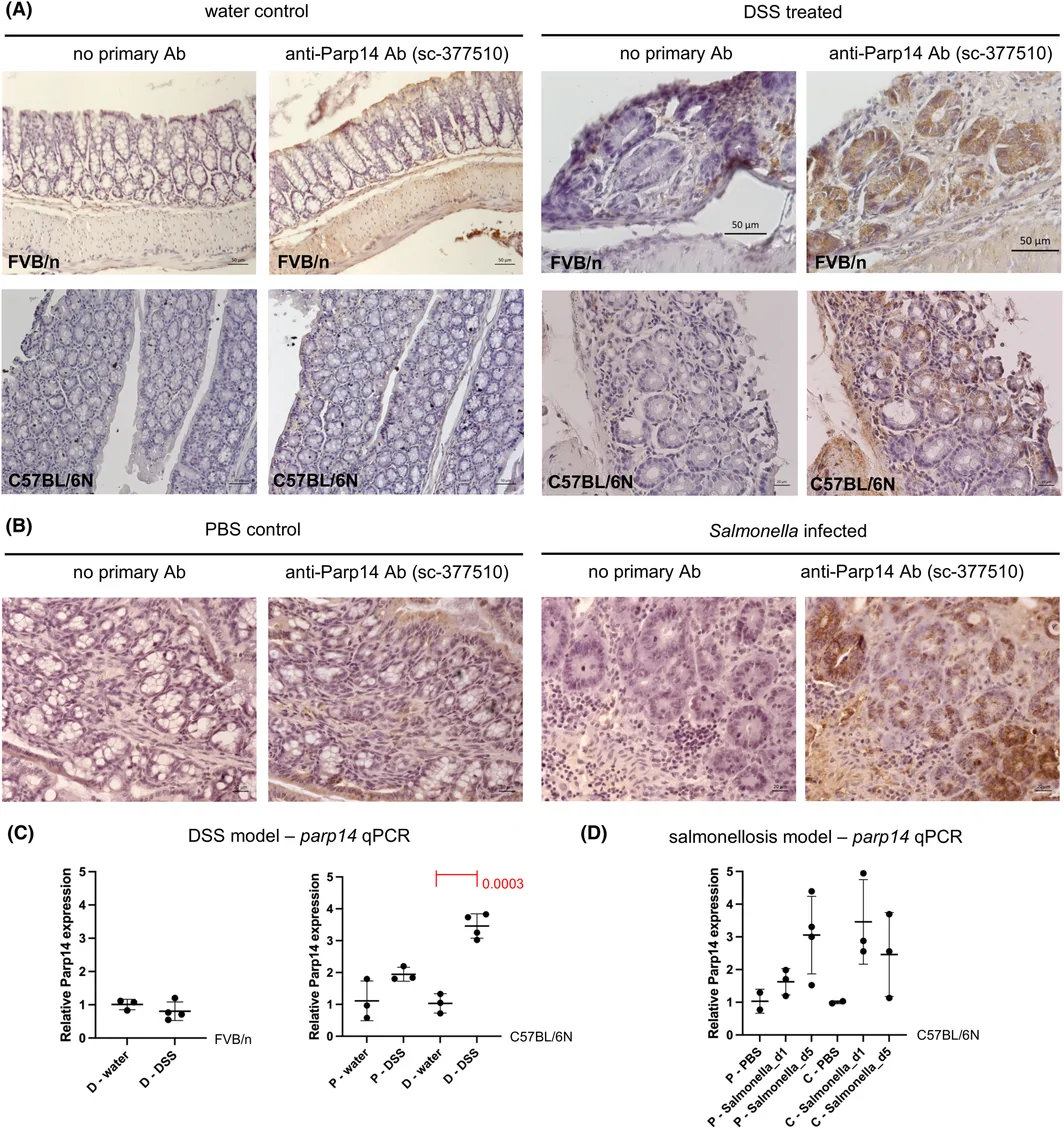
Parp14 expression and localization in the mouse colon. Parp14 expression was analyzed in two intestinal inflammation models—DSS and *Salmonella* colitis (Figure S4). (A) DSS colitis Parp14 IHC data. Parp14 IHC of the distal colon of water control and DSS (2.5%, 7 days) treated FVB/n and C57BL/6N mice. 20× objective images are shown (1:500 dilution of anti‐Parp14 antibody). (B) Salmonellosis Parp14 IHC data. Parp14 IHC of the proximal colon of PBS control and *Salmonella‐*infected (5 days) C57BL/6N mice. 40× objective images are shown (1:1000 dilution of anti‐Parp14 antibody). (C) DSS colitis (Figure S4A,B) and (D) salmonellosis (Figure S4C) qPCR data on relative *parp14* expression (means with standard deviation, statistics with two‐tailed unpaired *t*‐test). Samples were included in the data analysis if they passed the 0.5 standard deviation Ct filter for replicate runs (see Materials and Methods section). The calibrators in Figure 1C are the means of D‐water (FVB/n experiment, Figure S4A) and P‐water (C57BL/6N experiment, Figure S4B), and in Figure 1D, the mean of P‐PBS (Figure S4C). C, cecum; D, distal colon; P, proximal colon.

### 2.6 Quantitation of histological changes in the mouse colon tissue

FFPE tissues were cut into longitudinal, 5 μm thick sections prior to hematoxylin and eosin (HE) staining. HE staining was performed using standard methods. All samples were scanned using a Pannoramic 1000 Slide scanner (3DHistech, Budapest, Hungary) with a 20× objective and analyzed with a Pannoramic viewer (3D Histech, software version 1.15.4). The severity of colonic inflammation was scored according to Ref. [22]. Briefly, (i) immune cell infiltration: score of 0 = healthy, 1 = some inflammatory cells seen in mucous, 2 = moderate infiltration, and 3 = severe and acute inflammation; (ii) edema scoring was based on location and severity of edema and ranged from 0 to 3; (iii) erosion depth: 0 = no erosion, 1 = erosion of epithelium, 2 = erosion of mucous, 3 = erosion going through muscular lamina; (iv) loss of goblet cells: 0 = no change, 1 = weak, 2 = moderate, and 3 = severe effect/lot. Two people performed the scoring independently and blindly for the sample group. The presented scores are the averages of these two analyses. Variables such as edema, erosion, goblet cell loss, and immune infiltration were measured for the distal and proximal colon. Variable colon length was measured in millimeters (mm) and the means of measurements from each group were calculated. Variables were summarized with descriptive statistics, and associations between groups and variables were studied by the Kruskal–Wallis test and the Dwass–Steel–Chritchlow–Fligner test for post hoc comparisons. The normality of variables was evaluated visually and tested with the Shapiro–Wilk test. Due to the non‐normality of the continuous variables, nonparametric methods were used. The statistical significance level was set at .05 in all tests (two‐tailed). The analyses were performed using the SAS system, version 9.4 for Windows (SAS Institute Inc., Cary, NC, USA).

### 2.7 Mouse Parp14 immunohistochemistry

#### 2.7.1 Anti‐Parp14 IHC

Mice (C57BL/6N and FVB/n) FFPE tissue sections were stained with the anti‐Parp14 antibody in the same way as for human FFPE biopsy specimens, as mentioned above.

#### 2.7.2 Double immunofluorescence

Anti‐F4/80 (rat anti‐mouse MCA497A647, Biorad, dilution 1:500) and anti‐Parp14 (sc377150, Santa Cruz Biotechnology, dilution 1:500) were mixed in 5% (w/v) BSA in TBST and added to colon sections post‐blocking for overnight incubation at 4°C in a humidified chamber. The anti‐F4/80 antibody was Alexa‐fluor 647 conjugated. Secondary antibody Alexa‐fluor 488 goat anti‐mouse IgG (H+L) (A11001, Invitrogen, dilution 1:1000) was used to visualize mouse anti‐Parp14. Tissue sections were washed with PBST and counterstained using 300 nM of DAPI (sc‐3598, Santa Cruz) for 1 min. Mounting was done using Histo‐Clear, and sections were kept in the dark at 4°C before imaging using the Zeiss AxioImager M1. DAPI (nucleus), GFP (green, Parp14), and Alexa‐fluor 660 (red, F4/80) channels were used in image acquisition.

### 2.8 Isolation of total RNA and quantitative PCR analysis

Total RNA was isolated from mouse tissues using TRIsure reagent (BIO‐38033, Bioline Gmbh, Germany), and genomic DNA was digested using RNase‐free DNase (rDNase) from Machery‐Nagel as per the manufacturer's instructions. Briefly, colon tissue was homogenized using stainless steel balls (IKA 5 mm stainless steel balls, Fisher scientific) and a compact bead mill (TissueLyser LT, Qiagen). Colon tissue was placed in the TRIsure reagent during homogenization. After this, chloroform was used for phase separation. RNA in the upper aqueous phase was precipitated using isopropyl alcohol. The pellet was washed with 70% ethanol and dissolved in nuclease‐free water. Dissolved RNA was mixed with rDNase and rDNase reaction buffer as per the manufacturer's instructions. This mixture was incubated at 37°C for 10 min. Post‐gDNA digestion, RNA was precipitated using 3M sodium acetate, pH 5.2, and 96% ethanol. The pellet was washed with 70% ethanol and dissolved in RNase free water. RNA purity and concentration were measured using a DeNovix DS‐11 spectrophotometer (Wilmington, DE, USA). 1 μg of dissolved RNA was reverse transcribed using SuperScript III reverse transcriptase (#1808044, ThermoFisher Scientific) and Oligo (dT) 12–18 Primer (#18418012, ThermoFisher Scientific). Separate real‐time PCR (RT‐PCR) was carried out in duplicates using TaqMan gene expression assays (Applied Bio systems, Foster city, Ca) for *parp14* (Assay ID: Mm00520984_m1) and the reference gene, glyceraldehyde‐3‐phosphate dehydrogenase, *gapdh* (Assay ID: Mm99999915_g1) on the Rotor‐Gene Q real‐time PCR cycler (Qiagen). Thermal cycling conditions included an initial denaturation step at 95°C for 10 min followed by 40 cycles of 95°C for 15 s and 60°C for 1 min. Relative mRNA levels were determined using the 2^−ΔΔCT^ method with GAPDH as reference.^24^ If the standard deviation of duplicate Ct values from a sample was 0.5 or more, the results of those samples were not used for statistical analysis. Statistical analysis of 2^−ΔΔCT^ values was done using the unpaired *t* test (two‐tailed).

### 2.9 Bacterial community analysis of a mouse fecal sample

DNA from fecal pellets was extracted using the Machery‐Nagel NucleoSpin DNA Stool kit (reference no. 740472.250) using the manufacturer's protocol. The 16S rRNA gene amplicons were sequenced at the University of Illinois, Roy J. Carver Biotechnology Center, DNA Services Laboratory. In brief, the 16S rRNA gene amplicons were generated with the barcoded Full‐Length 16S rRNA gene primers (forward: AGRGTTYGATYMTGGCTCAG, reverse: RGYTACCTTGTTACGACTT) from PacBio and the 2× Roche KAPA HiFi Hot Start Ready Mix. Amplicons were converted to a library with the SMRTBell Express Template Prep kit 2.0. The pooled library was sequenced on 3 SMRTcells 8M on a PacBio Sequel IIe using the CCS sequencing mode and a 12 h movie time. A total of three DNA extractions and one PCR water control were also sequenced along with the samples. The bioinformatics analysis was performed using mothur (v1.48.0) software. The raw 16S rRNA data were processed as described in Ref. [25] with modifications needed for the PacBio sequence analysis. In brief, the fastq.info command, in which the PacBio parameter was set to be true, was used to convert the fastq files to fasta files. The command make.group was used to assign each sequence to its sample. Subsequently, the merge. files command was used to merge fasta files. The screen.seqs was used to remove the ambiguity and homopolymer (maximum eight) sequences. Sequences were aligned using the Silva reference database (v138), followed by the removal of potential chimeras. Subsequently, sequences were clustered at a 97% similarity level to create an operational taxonomic unit (OTU) abundance table. The representative sequences of each OTU were classified using a mothur‐compatible Ribosomal Database Project (v18). All the statistical analysis was performed using R software (v4.1.1; R Core Team, 2020). A total of 954 OTUs were obtained across the five wt and five knock‐out mutant mouse samples (File S2). The number of sequences in each sample ranged from 2058 to 24 673. Two blank DNA isolation controls produced 47 OTUs. The subsampling for the equal sequencing depth (2058 sequences per sample) resulted in 524 OTUs. Among the 47 OTUs in the blank DNA isolation controls, only one OTU (Otu0063) was found in the fecal pellet samples. The subsampling resulted in the removal of the potential contaminant Otu0063 from the fecal pellet samples. The raw sequencing data from PacBio‐based mouse microbiota profiling has been deposited in the NCBI (https://www.ncbi.nlm.nih.gov) BioProject database with accession number PRJNA977978.

### 2.10 Flow cytometry‐based leukocyte profiling

#### 2.10.1 Cell isolation

The mid‐colon was excised into 10 mL of ice‐cold PBS, and the surrounding fat tissue was removed. The colon was cut open longitudinally and rinsed in PBS to remove fecal content. Colon was further cut into 5–10 mm pieces and put into a 15 mL falcon tube with 5 mL prewarmed 2 mM EDTA in Hank's balanced salt solution (HBSS, Gibco) and shaken at 250 rpm for 15 min at 37°C. Tube was manually shaken vigorously, and the supernatant was removed while tissue segments were recovered with a colander. 5 mL of warm 2 mM EDTA in HBSS was added on the segments, incubated for 30 min at 37°C with shaking at 250 rpm, and then shaken vigorously by hand. The epithelial cell fractions were discarded, and the lamina propria tissues were digested in 5 mL of enzyme cocktail containing 1 mg/mL Collagenase VIII (Sigma C2139) and 10 μg/mL Dnase I (D5025) diluted in RPMI (Corning) with 1% Fetal Calf Serum (FCS, BioWest, S181B) at 250 rpm for 45 min at 37°C. Digestive enzyme activity was stopped by adding 5 mL of cold FACS buffer (PBS, 2% FCS, 1 mM EDTA). Digested tissue was then filtered through a 70 μm cell strainer, centrifuged at 1500 rpm for 5 min, and stained for flow cytometry analysis. From the blood sample, 100 μL was taken to a tube containing a drop (approximately 10 μL) of heparin (Heparin LEO 100 IU/mL, LEO Pharma). Samples were kept on ice, and erythrocytes were lysed with hypotonic treatment. First, 1 mL of 0.2% NaCl was added per sample, vortexed for up to 15 s, and then 1 mL of 1.6% NaCl was added. Cells were pelleted (1006 g, 1.5 min), and lysis was repeated a total of four times. Finally, cells were washed twice with 1 mL of PBS and filtered through silk with a 77 μm pore size. Spleens were transferred to PBS on ice. Spleens were minced through a metal mesh using the plunger of a 1 mL syringe. The mesh was washed with 1 mL of PBS, and the cell suspension was collected and pelleted (1006 g, 1.5 min). To remove red blood cells from suspension, cells were lysed with a NaCl treatment by first adding 0.2% NaCl, vortexing for 15 s, and then adding 1.6% NaCl. Cell suspension was pelleted (1006 g, 1.5 min) and washed with PBS. Finally, cells were eluted into PBS and filtered through silk with a 77 μm pore size.

#### 2.10.2 Flow cytometry

Cells were stained with Fixable Viability Dye eFluor 780 (eBioscience, 65‐0865) to label the dead cells. Unspecific binding to low‐affinity Fcreceptors was blocked by incubating the cells with an unconjugated CD16/32 antibody (BioXCell, clone 2.4G2). Cells were subsequently stained for 30 min at 4°C with antibodies diluted in the FACS buffer. Immune cell panel included CD45‐PerCP‐Cy5‐5 (Becton Dickinson (BD), #550994), CD4‐PE (BD, #553049), CD8‐Bv650 (BD, #563234), B220‐PE‐CF594 (BD, #562313), CD11b‐BB515 (BD, #564454), CD11c‐BV711 (BioLegend, #117349), Ly6c‐BV421 (BD, #562727), Ly6G‐BV510 (BioLegend, #127633), SiglegF‐A647 (BD, #562680), CD64‐BV786 (BD, #741024), and F4/80‐PE‐Cy7 (Invitrogen, #25‐4801‐82). Cells were washed with FACS buffer and fixed with 1% formaldehyde in PBS. Samples were acquired with the LSRFortessa flow cytometer with FACSDiVaTM version 8 software (Becton Dickinson), and data were analyzed with the FlowJo software (FlowJo LLC). The gating strategies for flow cytometric analyses are depicted in Figure S7A. The results were statistically analyzed in GraphPad Prism.

### 2.11 Mouse colon tissue bulk RNA‐Seq and data analysis

The total RNA of the distal colon tissue samples was extracted as described above. The experimental steps as described in 2.11.2.‐2.11.7. were performed by Novogene Co., Ltd (Cambridge, UK, https://www.novogene.com/eu‐en/).

#### 2.11.1 RNA quality

RNA integrity was assessed using the RNA Nano 6000 Assay Kit of the Bioanalyzer 2100 system (Agilent Technologies, CA, USA).

#### 2.11.2 Library preparation for transcriptome sequencing

Briefly, mRNA was purified from total RNA using poly‐T oligo‐attached magnetic beads. Fragmentation was carried out using divalent cations at elevated temperatures in the First Strand Synthesis Reaction Buffer (5×). First strand cDNA was synthesized using a random hexamer primer and M‐MuLV Reverse Transcriptase (RNase H‐). Second‐strand cDNA synthesis was subsequently performed using DNA Polymerase I and RNase H. Remaining overhangs were converted into blunt ends via exonuclease/polymerase activities. After adenylation of the 3′ ends of DNA fragments, adaptors with a hairpin loop structure were ligated to prepare for hybridization. To select cDNA fragments of preferentially 370–420 bp in length, the library fragments were purified with the AMPure XP system (Beckman Coulter, Beverly, USA). Then PCR was performed with Phusion High‐Fidelity DNA polymerase, Universal PCR primers, and Index (X) Primers. At last, PCR products were purified (AMPure XP system), and library quality was assessed on the Agilent Bioanalyzer 2100 system.

#### 2.11.3 Clustering and sequencing

The clustering of the index‐coded samples was performed on a cBot Cluster Generation System using TruSeq PE Cluster Kit v3‐cBot‐HS (Illumia) according to the manufacturer's instructions. After cluster generation, the library preparations were sequenced on an Illumina Novaseq platform, and 150‐bp paired‐end reads were generated.

#### 2.11.4 Data analysis—Quality control

Raw data (raw reads) in fastq format were first processed through Novogene's in‐house perl scripts. In this step, clean data (clean reads) were obtained by removing reads containing adapter, reads containing ploy‐N, and low‐quality reads from raw data.

#### 2.11.5 Data analysis—Reads mapping to the reference genome

Reference genome and gene model annotation files were downloaded from genome website directly. The index of the reference genome was built using Hisat2 v2.0.5, and paired‐end clean reads were aligned to the reference genome using Hisat2 v2.0.5. We selected Hisat2 as the mapping tool because Hisat2 can generate a database of splice junctions based on the gene model annotation file, resulting in a better mapping result than other non‐splice mapping tools.

#### 2.11.6 Data analysis—Quantification of gene expression level

The featureCounts v1.5.0‐p3 was used to count the read numbers mapped to each gene. Subsequently, Fragments Per Kilobase of transcript sequence per million base pairs sequenced (FPKM) of each gene were calculated based on the length of the gene and the read count mapped to this gene.

#### 2.11.7 Data analysis—Differential expression analysis

Differential expression analysis of two conditions/groups (two biological replicates per condition) was performed using the DESeq2 R package (1.20.0). DESeq2 provides statistical routines for determining differential expression in digital gene expression data using a model based on the negative binomial distribution. The resulting *p*‐values were adjusted using Benjamini and Hochberg's approach for controlling the false discovery rate. Genes with an adjusted *p*‐value <.05 found by DESeq2 were assigned as differentially expressed.

#### 2.11.8 Data analysis—GO and KEGG analyses

To analyze cellular and physiological associations of the differentially expressed genes (DEGs) or uniquely expressed genes (FPKM > 1), we performed the Gene Ontology (GO) enrichment at the GO consortium website (https://geneontology.org).^26^, ^27^, ^28^ In parallel, we performed the Kyoto Encyclopedia of Genes and Genomes (KEGG) pathway analysis^29^, ^30^, ^31^ with SRplot^32^ (http://www.bioinformatics.com.cn/srplot). Only those DEGs that passed our stringent in‐house filtering criteria were used in the GO/KEGG analyses, that is, upregulated genes, log2(FoldChange) > 1 and *p*_adj_ < .05; downregulated genes, log2(FoldChange) < −1 and *p*_adj_ < .05. The GO term annotation was done with the binomial test type using the Bonferroni correction for multiple testing. The raw bulk mouse tissue RNA‐Seq sequencing data have been deposited in the NCBI (https://www.ncbi.nlm.nih.gov) Gene Expression Omnibus (GEO) database with accession number GSE252812.

### 2.12 Human bulk colon tissue transcriptome analysis

Bulk human tissue transcriptome data with accession numbers E‐GEOD‐14580^33^ and E‐GEOD‐4183^34^ were accessed from the ArrayExpress database (https://www.ebi.ac.uk/biostudies/arrayexpress) and biogps.org gene annotation portal.^35^ Using biogps.org, the Parp14 mRNA expression value (fluorescence intensity) for each patient from the two datasets was downloaded. Using sample information from ArrayExpress, sample groups were assigned, and the mean of the expression value was calculated to draw two separate graphs. The difference between the two groups was statistically measured using the Mann–Whitney test.

## 3 RESULTS

### 3.1 Parp14 is expressed by human colon epithelial cells and some cells in the lamina propria

To examine Parp14 expression and localization in the human colon, we analyzed biobank‐archived FFPE endoscopic biopsies (File S1). The patients had histologically been diagnosed either with UC (*n* = 12) or CD (*n* = 7). In addition, we analyzed the colon biopsies of patients with colonoscopy‐justifying symptoms (*n* = 9), but with a normal colon status upon histological scoring by a pathologist. Some cells in the lamina propria were positive for Parp14 (Figure 1A–C), as judged by the staining with a commercial mouse monoclonal anti‐Parp14 antibody (Figure S1). However, the staining was most evident in the cryptal epithelial cells or in the epithelial cells facing the lumen of the colon (hereafter surface epithelial cells). We also observed that Parp14 was mostly localized in the cytoplasm, and staining was granular both on the surface and in the cryptal epithelial cells (Figure S2). There were some crypts, in particular in UC patients, at the immune cell‐infiltrated sites with a thin mucous lining and lacking Goblet cells, which contained epithelial cells highly positive for Parp14 (Figure 1B). The Parp14 staining intensity was visually scored for two categories, that is, surface epithelial cells and cryptal epithelial cells (0, faint or negligible staining to 3, high intensity). The biopsy sections showed stronger Parp14 staining in the surface epithelial cells when compared to cryptal epithelial cells in all patient groups (Figure 1C,D). There was a trend toward less pronounced Parp14 staining in the surface epithelial cells of CD patients as compared to normal patients (*p*‐value .0851). However, statistically significant differences (*p*‐value <.05) in Parp14 staining intensity between normal, UC, and CD patients were not observed (Figure 1D). In the context of cell cultures, we witnessed that Parp14 expression was at a negligible basal level in HeLa229 cells, that is, in a human epithelial cell culture model (Figure S3). However, upon incubation with common inflammatory stimuli IFN‐α or IFN‐γ, more Parp14 was detected in a time‐dependent manner. Similar effects were also evident with THP‐1 cells, that is, in a human macrophage cell culture model (Figure S3). Taken together, Parp14 is expressed in the human colon, in particular by the epithelial cells, with a characteristic granular staining pattern in the cytosol.

### 3.2 Parp14 is expressed by mouse colon epithelial cells and macrophages in the lamina propria

To examine Parp14 expression and localization in the mouse colon, we first analyzed FFPE distal colon sections derived from the DSS model executed in two genetic backgrounds—FVB/n and C57BL/6N (Figure S4). In the water control FVB/n mice, Parp14 staining was faint, whereas in the DSS‐treated FVB/n mice (7 days, 2.5% DSS in drinking water), the colon crypt epithelial cells were strongly Parp14 positive (Figures 2A and S5). The staining was mostly granular in the cytoplasm, similar to the human colon (Figure S2). The DSS‐increased Parp14 staining was also evident, but not so clear, in the C57BL/6N background (Figure 2A). Other cell types in the mouse colon also expressed Parp14. Some Parp14 positive cells in the colon wall of DSS‐treated C57BL/6N mice were positive for the macrophage marker F4/80 (Figure S6). Next, we analyzed FFPE proximal colon sections derived from the salmonellosis C57BL/6N model (Figure S4). In the PBS control C57BL/6N mice, Parp14 staining was faint, whereas in the *Salmonella*‐infected mice (5 days post‐infection), the crypt epithelial cells of the proximal colon were strongly Parp14 positive (Figure 2B). The staining was granular in the cytoplasm. Parp14 expression was also quantified at the transcriptional level with bulk tissue samples. The level of Parp14 mRNA did not differ in the distal colon samples of water and DSS‐treated FVB/n mice (Figure 2C). However, Parp14 mRNA level was higher in the distal colon samples of DSS‐treated C57BL/6N mice as compared to water controls (Figure 2C). Moreover, Parp14 expression in the DSS‐treated C57BL/6N mice was higher in the distal colon as compared to the proximal colon (Figure 2C, *p*‐value .0018). *Salmonella* infection (1 and 5 days post‐infection) also resulted in higher *parp14* mRNA levels as compared to PBS control in the proximal colon and cecum of C57BL/6N mice (Figure 2D), but these differences were not statistically significant (lowest *p*‐value .084, cecum PBS vs. day 1 infection). Taken together, Parp14 is expressed in the proximal and distal mouse colon, in particular by the epithelial cells, based on experimentation in salmonellosis and DSS colitis models.

### 3.3 Parp14‐deficient mice have increased sensitivity to DSS colitis

To analyze the physiological functions of Parp14 in colitis, we used the body‐wide Parp14‐deficient mice^23^ backcrossed in‐house to C57BL/6N background (Figure S7). Littermates of wt and Parp14‐deficient mice were treated with DSS and water as a control, as described in Figure 3A. We monitored the mice daily by quantifying their body weight, stool consistency, and amount of blood in the stool. Oral administration of DSS caused a loss in body weight in both wt and Parp14‐deficient mice (Figure 3B) in a statistically similar manner. The DSS‐treated Parp14‐deficient mice did not differ from wt mice in stool consistency (Figure 3C). However, the Parp14‐deficient mice suffered from increased rectal bleeding (Figure 3D) in comparison to wt mice upon DSS treatment. This phenotype trend appeared on day 4, but it took until day 8 to reach statistical significance. The DSS treatment caused a reduction in colon length to a similar extent in wt and Parp14‐deficient mice (Figure 3E). We also performed a histopathological analysis of the distal and proximal regions of the colon. The H&E‐stained FFPE sections were scored for edema, epithelial erosion, Goblet cell loss, and immune cell infiltration. The absence of Parp14 did not cause apparent tissue damage in the water‐control mice, but it worsened the overall tissue damage observed upon the DSS treatment (Figure 3F). Scoring of the different pathological variables revealed significantly stronger epithelial erosion and more pronounced Goblet cell loss as well as immune cell infiltration in the distal colon of Parp14‐deficient mice as compared to wt mice (Figure 3G). Also, the Parp14‐deficient mice suffered from more pronounced Goblet cell loss in the proximal colon (Figure 3G). The body‐wide genetic deficiency of Parp14 sensitized mice to DSS colitis.

**FIGURE 3:**
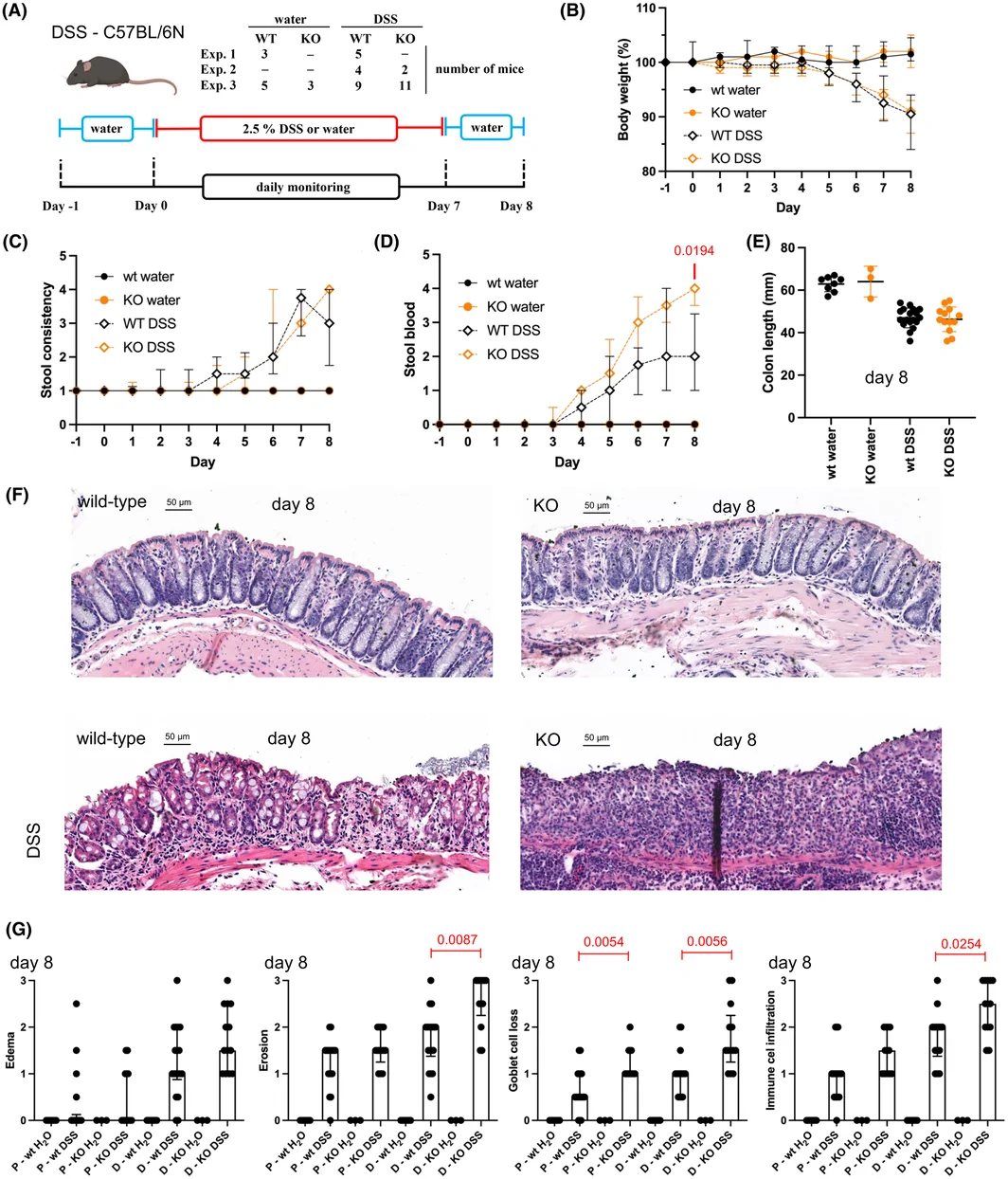
Body‐wide genetic deficiency of Parp14 sensitizes mice to DSS colitis. (A) Schematic description of the DSS mouse experiments. Animals from three different experiments were pooled for the analyses. (B) Weight change of the mice during the course of the experiments relative to day 1 (medians with interquartile range). No statistical differences between wt and Parp14‐deficient mice were detected. (C) Stool consistency scoring (0—normal feces to 4—loose stool/diarrhea, medians with interquartile range). No statistical differences between wt and Parp14‐deficient mice were detected. (D) Stool scoring for blood (0—no blood to 4—bloody diarrhea with rectal bleeding, medians with interquartile range). A statistically significant difference between wt and Parp14‐deficient mice is indicated in the figure. (E) Colon length of the sacrificed mice (means with standard deviation). No statistical differences between wt and Parp14‐deficient mice were detected. (F) Images of the H&E‐stained FFPE sections of distal colon samples of water and DSS treated wt and Parp14‐deficient mice. 20× objective images are shown. (G) Quantitation of pathological variables, that is, tissue edema, epithelial erosion, Goblet cell loss, and immune cell infiltration in the proximal and distal colon (medians with interquartile range). Statistical differences between wt and Parp14‐deficient mice are indicated in the figure. The statistical analyses are described in the methods section.

### 3.4 Parp14‐deficient mice have normal fecal bacterial microbiota

Bacterial colon dysbiosis in the Parp14‐deficient mice could have contributed to their increased sensitivity to DSS colitis. To explore this possibility, we used PacBio long‐read sequencing of the nearly entire 16S rRNA gene and characterized the fecal bacterial microbiota during routine in‐house colony maintenance. We used the OTU approach with the canonical 97% sequence similarity cutoff, that is, raw reads that differed by <3% were clustered into a single OTU sequence. The taxonomy resolution of the identified OTUs (File S2) is shown in Figure 4A. Overall, the numbers of the identified taxa at different taxonomic levels did not strongly differ between the wt and Parp14‐deficient mice. The % relative abundance heatmaps of the identified top 40 genera and top 40 species are shown in Figure 4B. To gain deeper statistical insights with the sub‐sampled dataset, we first compared the Chao1 and Shannon alpha‐diversity indexes. There were no statistically significant differences observed in Chao1 and Shannon alpha‐diversity indexes between wt and Parp14‐deficient mice (Figure 4C), that is, the OTU richness in the samples did not differ. Secondly, to study if the bacterial community compositions differed between wt and Parp14‐deficient mice, we used principal coordinate analysis (Figure 4D) as well as permutational analysis of variance and beta dispersion analysis with Bray–Curtis dissimilarity metrics (data not shown). These beta diversity analyses demonstrated that the bacterial community OTU composition of wt and Parp14‐deficient mice did not statistically differ. Taken together, the absence of Parp14 does not appear to cause colon bacterial dysbiosis in Parp14‐deficient mice during routine in‐house colony maintenance.

**FIGURE 4:**
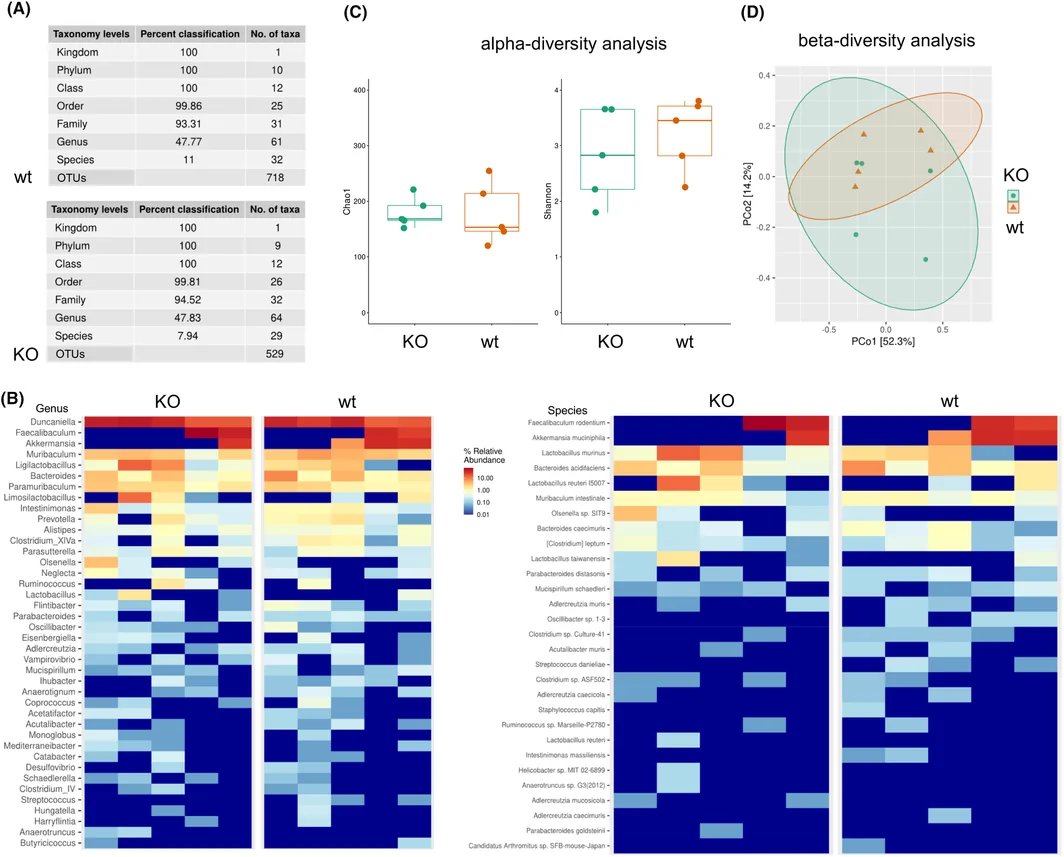
Body‐wide genetic deficiency of Parp14 does not affect the fecal bacterial microbiota. (A) The taxonomy resolution of the identified 16S rRNA OTUs (File S2). The PacBio sequence reads of the nearly entire 16S RNA gene were clustered into a single OTU based on the canonical 97% sequence homology cut‐off. (B) Heat maps showing the fecal bacterial microbiota profile of wt and Parp14‐deficient mice at the genus (left) and species (right) levels. A total of 40 most abundant taxa are listed from top to bottom in decreasing order of % relative abundance (taxa‐specific OTU reads/all reads in a sample × 100). Each column represents a single individual mouse sample. (C) The Chao1 and Shannon alpha‐diversity indexes of the microbiota OTU richness differences between wt and Parp14‐deficient mice. Each point represents a single individual mouse sample. Statistical differences between the OTU richness values of wt and Parp14‐deficient mice were not detected. (D) The beta‐diversity principal coordinate analysis of the microbiota OTU compositional differences between wt and Parp14‐deficient mice. The ellipses were drawn based on the 95% confidence intervals. Each point represents a single individual mouse sample. Differential clustering of the data points of wt and Parp14‐defient mice was not detected. Also, permutational analysis of variance and beta dispersion analysis with Bray–Curtis dissimilarity metrics (data not shown) did not reveal microbiota OTU compositional differences between wt and Parp14‐deficient mice.

### 3.5 Parp14‐deficient mice have normal colon leukocyte populations

Abnormal colon immune cell population structure in Parp14‐deficient mice could contribute to their increased sensitivity to DSS colitis. We used flow cytometry to detect different leukocytes from the mid‐colon, blood, and spleen post‐DSS and water control treatments (Figures 5 and S8). We stained single‐cell suspensions for cells of the myeloid lineage, that is, granulocytes (neutrophils and eosinophils), monocytes, macrophages and dendritic cells, as well as for cells of the lymphoid lineage (B and T cells). We did not detect significant differences in any of the leukocyte populations between water‐treated wt and Parp14‐deficient mice. The data implies that Parp14 is not a substantial driver of leukocyte development/homeostasis in mice (Figures 5 and S8). In both genetic backgrounds and to a similar extent, the DSS treatment increased the proportion of neutrophils and classical Ly6C^Hi^ and non‐classical Ly6C^Low^ monocytes in the mid‐colon compared to water‐treated controls (Figure 5). In a similar manner, DSS treatment significantly decreased the proportion of CD11c dendritic cells and the proportion of F4/80 macrophages (Figure 5). Some genotype differences were evident in the blood and spleen. In the blood of DSS‐treated Parp14‐deficient mice, the proportion of circulating classical Ly6C^Hi^ monocytes was decreased as compared to wt mice (*p*‐value .0322) (Figure S8). Also, DSS did not induce a change in the proportion of circulatory CD8 T cells in the Parp14‐deficient mice (*p*‐value .5457), although this took place in the wt mice (*p*‐value of .0430) (Figure S8). Furthermore, the absence of Parp14 caused a DSS‐associated increase in the proportion of circulatory CD4 T cells (*p*‐value .0242), which was not evidenced in the wt mice (*p*‐value .6232) (Figure S8). In the spleen, a lower proportion of CD8 T cells (*p*‐value .0205) was detected in the DSS‐treated Parp14‐deficient mice as compared to the water control, while in the wt mice, this effect was not detected (*p*‐value .3644) (Figure S8). Overall, the data indicate that the DSS‐treated Parp14‐deficient mice had abnormal numbers of monocytes and T cells in the blood and spleen. The colon leukocyte populations of Parp14‐deficient mice appeared normal.

**FIGURE 5:**
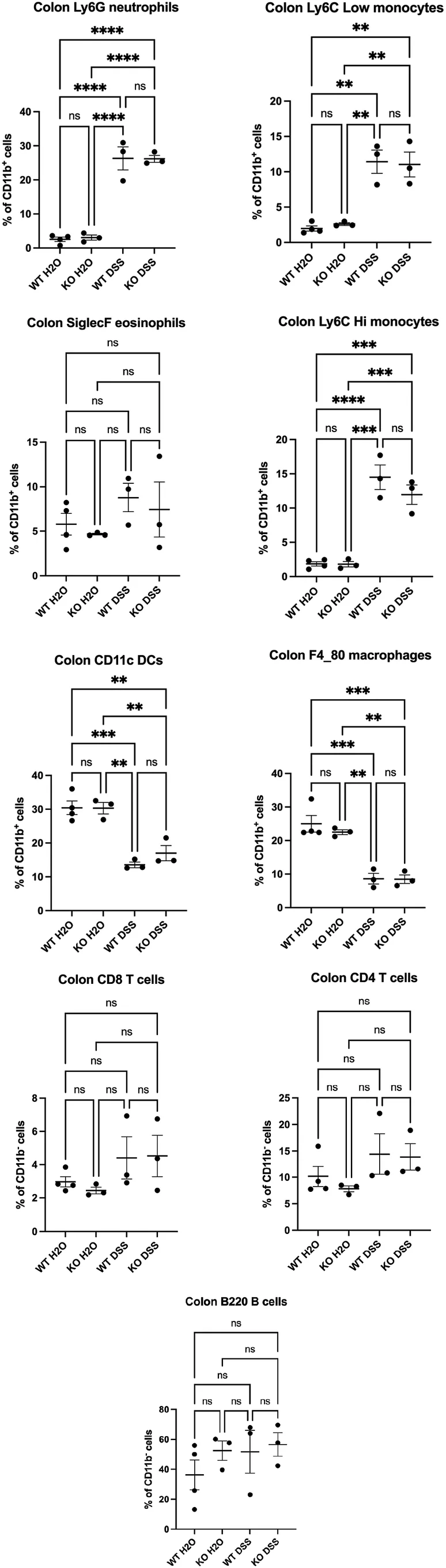
Body‐wide genetic deficiency of Parp14 does not affect the colon leukocyte populations. Mid‐colon single‐cell suspensions post‐DSS and control treatment at day 8 were stained for myeloid‐derived cells, that is, granulocytes (neutrophils and eosinophils), monocytes, macrophages, dendritic cells, and lymphoid‐derived cells (B and T cells). Each dot represents one mouse from DSS mouse experiment 3 (Exp 3, Figure 3A). Refer to the gating strategy in Figure S8. Data are presented as mean ± SEM. Statistical analysis was done with a one‐way ANOVA with the Bonferroni post‐hoc test. (*p*‐values ‐ ns, > 0.05; **, < 0.01; ***, < 0.001; ****, < 0.0001).

### 3.6 Parp14‐deficient mice have an abnormal colon transcriptome after DSS stress

Abnormal functions of cells in the colon wall could explain the increased sensitivity of Parp14‐deficient mice to DSS colitis. To obtain transcriptomes as a proxy of cellular functions, we performed a bulk tissue RNA‐Seq analysis of the mouse colon distal sections post‐DSS and post‐water control treatments on day 8. The key reliability and descriptive metrics are shown in Figure S9A–C. Numbers of genes detected to be expressed (FPKM > 1), as well as the differential gene expression metrics, are shown in Figure S9D,E and File S3. Of note, Parp14 was upregulated in the wt mice post‐DSS treatment (1606 log2FoldChange, 3.044‐fold up in linear scale, *p*_adj_ .003). Based on the DEGs (File S3), we performed GO term (Figure 6A‐E) and KEGG pathway (Figure 6F‐I) analyses. Only those DEGs that passed stringent filtering criteria were used, that is, upregulated genes, log2(FoldChange) > 1 and *p*_adj_ < .05; downregulated genes, log2(FoldChange) < −1 and *p*_adj_ < .05 (File S3). Considerable differences were detected between wt and Parp14‐deficient mice. The upregulated DSS response in wt mice was dominated by DNA replication‐ and cell division‐related GO terms and KEGG pathways (Figure 6B,F, File S4). Inflammation‐ and infection‐related GO terms and KEGG pathways were also identified, but they appeared relatively low in the ranking. In contrast, the upregulated DSS response of Parp14‐deficient mice was dominated by inflammation‐ and infection‐related GO terms and KEGG pathways (Figure 6C,G, File S4). In respect of the GO terms, all the top 15 scoring terms were related to inflammation and infection (Figure 6C), and 11 of these were not detected at all in the upregulated DSS response of the wt mice. It is remarkable that, in comparison to the wt mice, the downregulated DSS response of Parp14‐deficient mice was scarce in the number of GO term and KEGG pathway identifications (Figure 6D,E,H,I, File S4). Taken together, Parp14‐deficient mice had an abnormal transcriptome after the DSS stress, dominated by genes with functions in inflammation and infection responses.

**FIGURE 6:**
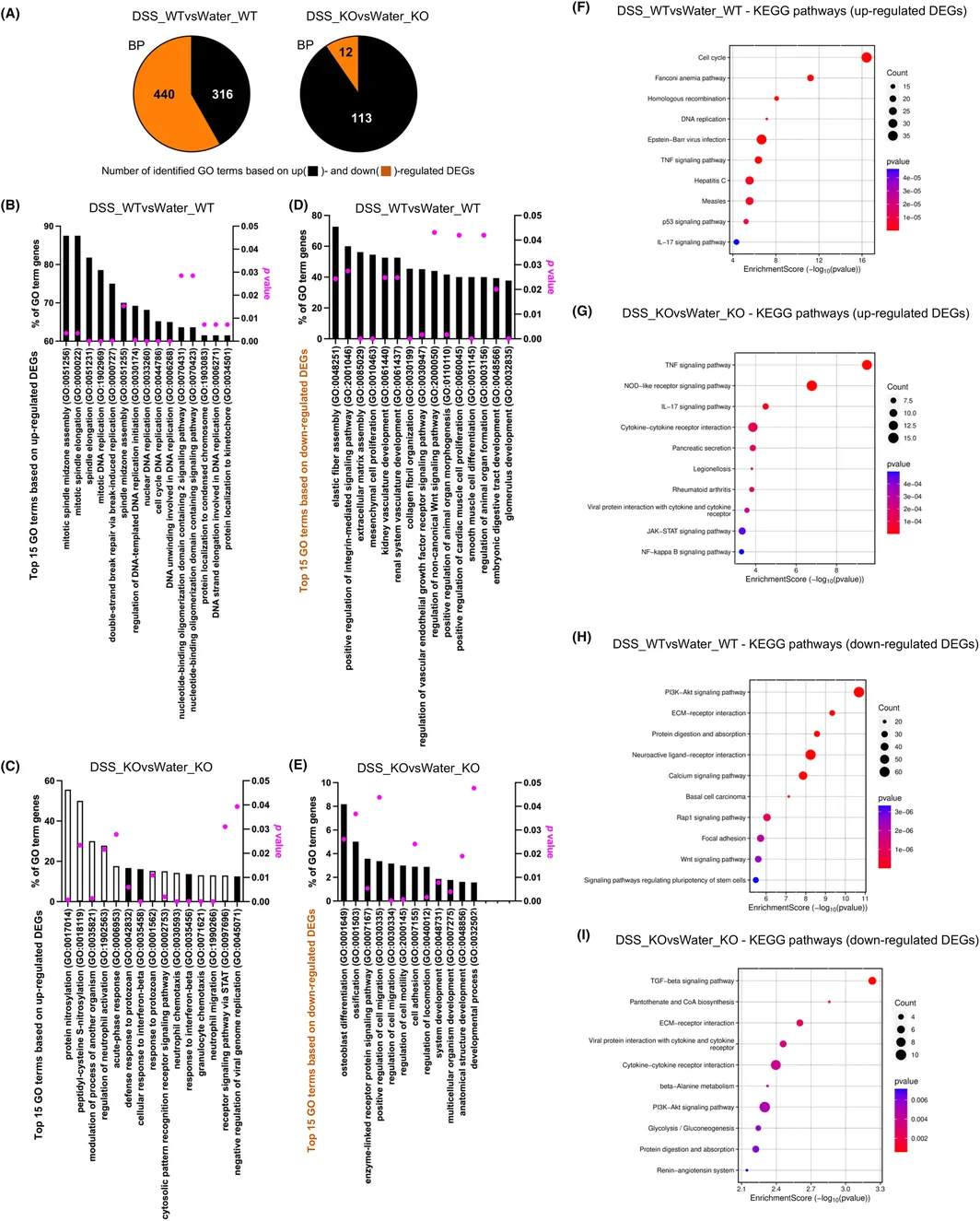
Parp14‐deficient mice have abnormal transcriptomic response to DSS stress. Data of a bulk tissue RNA‐Seq analysis of the mouse colon distal sections post‐DSS versus post‐water control treatment at day 8 are displayed (DSS mouse experiment 3, Exp 3, Figure 3A). (A) GO term analysis with genes that were differentially expressed in the DSS versus water treated wt as well as in the DSS versus water treated Parp14‐deficient mice. Based on the DEGs (File S3), GO biological process (BP) terms were searched with binomial test using the Bonferroni correction. (B–E) Bar graph representations of the top 15 identified GO BP terms (all the 12 identified GO BP terms in E) sorted based on the % of GO term genes‐values (number of detected genes in a particular GO term/number of all genes in a particular GO term × 100). The white bars refer to the unique DSS response of the Parp14‐deficient mice. All the identified GO PB terms with the corresponding gene lists are described in File S4. (F–I) Pathway enrichment dot blot representations of the top 10 identified KEGG pathways sorted based on the *p*‐value. All the identified KEGG pathways with the corresponding gene lists are described in File S4. The count values refer to the number of genes that were detected in a particular KEGG pathway.

### 3.7 Parp14‐deficient mice have an abnormal colon transcriptome in the resting state

Next, we analyzed if Parp14‐deficiency had already caused transcriptional changes in the colon prior to DSS exposure. Therefore, we analyzed the DEGs detected in the water‐treated Parp14‐deficient versus wt mice at day 8. Based on the genes upregulated in Parp14‐deficient mice (*n* = 106), we did not identify any GO terms (Figure 7A, File S4). In contrast, based on the genes downregulated in Parp14‐deficient mice (*n* = 78), we identified 12 GO terms (11 BP, 1 CC, and 0 MF), which were all related to inflammation or infection (Figure 7A,B, File S4). Out of all the 78 downregulated genes, 34 (44%) were mapped to these 12 GO terms (Figure 7C). KEGG pathway analysis further indicated that the genes downregulated in Parp14‐deficient mice were frequently categorized to inflammation and infection‐related cellular functions (Figure 7D,E, File S4). We also analyzed genes detected to be expressed in the water‐treated wt mice (mean FPKM > 1), not at all in the Parp14‐deficient mice (mean FPKM < 1) (Figure S9D). Based on the KEGG analysis, 51 genes out of the 639 such wt genes identified pathways with frequent functional relevance to inflammation and infection (Figure S10, File S5). Taken together, the Parp14‐deficient mice had an abnormal colon transcriptome in the resting state, dominated by genes with functions in inflammation and infection responses.

**FIGURE 7:**
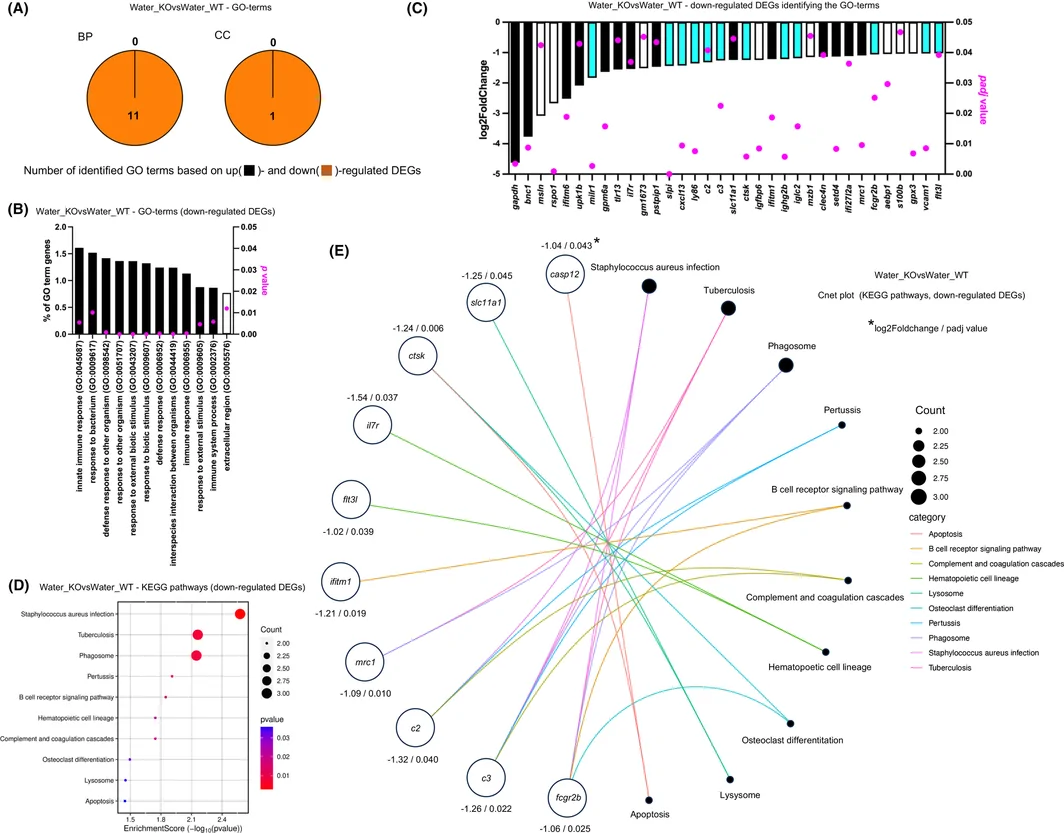
Parp14‐deficient mice have an abnormal colon transcriptome in the resting state. Data from a bulk tissue RNA‐Seq analysis of the mouse colon distal sections of water‐treated wt and Parp14‐deficient mice are displayed (DSS mouse experiment 3, Exp 3, Figure 3A). (A) GO term analysis with genes that were differentially expressed in the water‐treated Parp14‐deficient mice versus the wt mice. Based on the DEGs (File S3), GO terms (biological process, BP; molecular function, MF; cellular component, CC) were searched with a binomial test using the Bonferroni correction. No MF terms were identified. (B) Bar graph representation of all the identified GO terms sorted based on the % of GO term genes‐values (number of detected genes in a particular GO term/number of all genes in a particular GO term × 100). The white bar refers to the single identified GO‐CC term. All the identified GO PB terms with the corresponding gene lists are described in File S4. (C) All the downregulated genes leading to the identification of the 12 GO terms (B). Out of all the 78 downregulated genes (Water_KO vs. Water_WT), 34 (44%) were mapped to these 12 GO terms. The white bars refer to the DEGs leading uniquely to this one CC GO term identification. The cyan bars refer to the DEGs leading both to this one CC and the 11 BP GO term identifications. The black bars refer to the DEGs leading uniquely to the 11 BP GO term identification. (D) Pathway enrichment dot blot representation of the top 10 identified KEGG pathways with down‐regulated DEGs (Water_KOvsWater_WT) sorted based on the *p*‐value. The count values refer to the number of genes that were detected in a particular KEGG pathway. (E) The Cnetplot representation of the top 10 identified KEGG pathways with downregulated DEGs (Water_KO vs. Water_WT). The lines connect the KEGG pathways with the corresponding DEGs. All the identified KEGG pathways with the corresponding gene lists are described in File S4. The count values refer to the number of genes that were detected in a particular KEGG pathway.

## 4 DISCUSSION

Parp14 is an important regulator of immune cell functions, in particular, in lymphocytes and monocyte/macrophages.^7^, ^8^, ^9^, ^10^, ^11^, ^12^, ^13^, ^14^, ^15^, ^16^ We explored the effect of body‐wide genetic Parp14deficiency in the murine model of IBD, that is, in the 1‐week oral DSS exposure colitis model.^17^, ^18^ We also conducted a histological survey of Parp14 expression and localization in the mouse colon, in the DSS colitis model, and, in parallel, in the oral *Salmonella* exposure colitis model,^19^ and in the human colon using FFPE colon biopsies of IBD patients. The data highlight Parp14 as a protein highly expressed by epithelial cells, and having an important role in the maintenance of colon epithelial barrier integrity.

The histological inspection of our IBD patient biopsies (UC, *n* = 11; CD, *n* = 7; control, *n* = 9) demonstrated that Parp14 staining was most evident in the cryptal epithelial cells and in the epithelial cells facing the lumen of the colon, that is, surface epithelial cells. We also observed that Parp14 was mostly localized in the cytoplasm, and the staining was granular. The granular staining pattern might be related to Parp14 localization into processing bodies (P‐bodies), as reported with in vitro co‐localization analysis of endogenous DEAD‐box helicase 6 (DDX6) P‐body marker and transfected GFP‐Parp14 in HEK293T cells.^36^ Overall, more intense Parp14 staining was detected on the surface as compared to the cryptal epithelial cells in all three patient groups. This staining pattern could be related to the fact that the surface epithelial cells are more exposed to the colon luminal content, including the microbiota. Statistically significant differences were not detected in Parp14 staining intensity between the three patient groups. However, there were some crypts, particularly in UC patients, at the immune cell‐infiltrated sites with a thin mucous lining and lacking Goblet cells, which contained epithelial cells highly positive for Parp14. This could be related, in addition to luminal content assault, to stimulus from the cells of the lamina propria, for example, to the Parp14 upregulating interferons^15^ (see Figure S3). While our study was in progress, a bulk colon RNA‐Seq‐driven study was published implying *parp14* as a potential driver gene in UC progression from limited disease to extended disease.^37^ Histologically, the frequency of lamina propria cells with high Parp14 nuclear staining (rabbit polyclonal anti‐Parp14, ab224352, epitope unknown), some of which were CD68‐positive macrophages, was significantly higher in patients with UC that subsequently extended (*n* = 6) as compared with non‐extenders (*n* = 5).^37^ This nuclear localization of Parp14 most likely reflects its potent role as a transcriptional regulator.^7^, ^8^, ^15^ We also detected Parp14‐positive lamina propria cells, but based on our cohort and the mouse monoclonal anti‐Parp14 antibody (see Figure S1), the staining intensity in the cytosol of epithelial cells dominated the overall histology. Of note, the histology images of Argmann and co‐workers^37^ also reveal granular cytosolic Parp14 staining in the human colon epithelial cells, but this finding was not addressed in their study. More recently, a bioinformatics‐driven study on publicly available transcriptome data identified *parp14* as a ferroptosis‐related gene upregulated in UC.^38^ We also found highly significant upregulation of *parp14* in two public repository UC and CD IBD cohort transcriptomes^33^, ^34^ (Figure S11). Overall, Papr14 appears as a possible new mRNA or protein‐based colon biopsy biomarker in IBD. More elaborate cohorts should be executed to evaluate its prognostic and predictive potential in IBD.

To analyze the physiological functions of Parp14 in colitis we used the body‐wide Parp14‐deficient mice^23^ backcrossed in‐house to C57BL/6N background. Oral administration of DSS to mice via drinking water induces severe colitis characterized by weight loss, bloody diarrhea, loss of epithelial cells, and neutrophil infiltration, resembling flares in human UC.^17^, ^18^ DSS is believed to cause direct damage to the epithelial cells, followed by strong immune system activation by dissemination of the proinflammatory colon luminal content, such as bacteria, into the sub‐epithelial space.^17^, ^18^ Most likely, the strong pro‐inflammatory response, including oxidative stress, exacerbates the deleterious effects of DSS. Our key human histological findings were replicated in the DSS and also in *Salmonella* colitis models, in particular the strong granular cytosolic Parp14 staining in the cryptal epithelial cells of an inflamed colon. Also, based on the DSS model, some of the Parp14 positive mouse lamina propria cells were F4/80‐positive and thereby classified as macrophages. As compared to wt mice, Parp14‐deficient mice displayed increased rectal bleeding as well as stronger epithelial erosion, Goblet cell loss, and immune cell infiltration, in particular, in the distal colon upon DSS exposure. Extreme epithelial erosion was frequently evidenced in the Parp14‐deficient mice (see Figure 3F). The body‐wide genetic deficiency of Parp14 in C57BL/6N background sensitized mice to DSS colitis.

Abnormal colon microbiota or abnormal colon immune cell population structure could explain the increased sensitivity of Parp14‐deficient mice to DSS colitis. We did not detect differences in the fecal microbiota composition between the wt and Parp14‐deficient mice during our routine colony breeding. Therefore, it appears unlikely that the pro‐inflammatory stimulus of a differential colon microbiota explains the observed phenotypes upon DSS exposure. Similarly, we did not detect differences in the colon monocyte/macrophage, dendritic cell, eosinophil, neutrophil, B cell, and helper as well as killer T cell populations between the wt and Parp14‐deficient mice after the DSS exposure or control water treatment. In respect of neutrophils, these data were somewhat unexpected, as more pronounced immune cell (neutrophil) infiltration was scored for Parp14‐deficient mice under DSS exposure with colon histological sections. This discrepancy could be explained by the mid (leukocyte flow cytometry) versus distal (H&E histology) locations of the analyzed samples. Unfortunately, the scarcity of sample material did not allow us to conduct flow cytometry‐based leukocyte profiling of the distal colon. It is noteworthy that some leukocyte population structure differences between the wt and Parp14‐deficient mice were detected in the peripheral tissues, namely in the spleen and blood monocyte/macrophage, T cell, and B cell populations. In respect of lymphocytes, these data might reflect the early findings of T and B cell sub‐population abnormalities in Parp14‐deficient mice, for example, lower marginal zone and higher follicular splenic B cell proportion as compared to the wt littermates.^23^ However, the statistically significant effects detected in our study were always in the same direction as evidenced in the other genotype (see Figure S8). For example, in the blood of DSS‐treated Parp14‐deficient mice, the proportion of circulating classical Ly6C^Hi^ monocytes was decreased as compared to wt mice. Yet, a similar kind of trend without statistical support was already apparent in the control water‐treated mice. We think that more experimentation with bigger numbers of mice would be needed to substantiate these findings. Moreover, it is the colon leukocyte population structure that intuitively should have the major impact, if any, on the observed phenotypes upon DSS exposure. Based on the data, such differences do not exist between wt and Parp14‐deficient mice, not upon DSS exposure or control water treatment. The data imply that Parp14 is not a substantial driver of leukocyte development/homeostasis in mice and that Parp14 does not affect the mouse colon leukocyte population structure upon DSS exposure.

The increased sensitivity of Parp14‐deficient mice to DSS colitis could be explained by functional abnormalities of the different colon cells. To experimentally address this possibility, we determined transcriptomes as a proxy of cellular functions using bulk distal colon tissue RNA‐Seq. We clustered DEGs to cellular and physiological functions by GO term and KEGG pathway analyses. Considerable differences between the DSS exposure‐associated GO terms and KEGG pathways were detected. First, the upregulated DSS response in wt mice was dominated by DNA replication‐ and cell division‐related GO terms. This implies that the wt mice were more or less coping with the DSS exposure by enhancing colon wall renewal. Inflammation‐ and infection‐related GOs/KEGGs were also identified, but in the ranking, they appeared low. In contrast, the upregulated DSS response of Parp14‐deficient mice was dominated by inflammation‐ and infection‐related GOs/KEGGs. All the top 15 scoring GO terms were related to inflammation and infection, and 11 of these were not detected at all in the wt mice. This implies, in accordance with our disease severity scores, that the Parp14‐deficient mice were more or less beyond the epithelial renewal attempts while trying to merely cope with the strong sub‐epithelial assault of the luminal content. However, it remains elusive if the detected transcriptomic profile in the Parp14‐deficient mice was driving the pathology, that is, the more severe DSS colitis, or if it merely reflected it. Based on in vitro experimentation with macrophages, Parp14‐deficiency induces the pro‐inflammatory M1 polarization upon IFN‐γ stimulation, whereas, at the same time, it suppresses the anti‐inflammatory M2 polarization upon IL‐4 stimulation.^13^ It is tempting to speculate that Parp14‐deficiency in the macrophages has converted the colon wall into a more tissue‐damaging environment under DSS exposure. However, our data demonstrated high Parp14 expression and, thereby, highly plausible functions in the epithelial cells. As our bulk tissue RNA‐Seq data lack cell identity information both spatially and temporally, single‐cell spatial transcriptomics could in the future provide a better understanding of Parp14 functions. This information could also allow the creation of hypotheses for feasible cell culture‐based experiments to understand at the molecular level how and in which cell type Parp14 executes its functions.

We hypothesized that Parp14‐deficiency had already caused transcriptional changes in the colon prior to DSS exposure. To experimentally address this possibility, we compared the water treated Parp14‐deficient mice to water‐treated wt mice. A relatively low number of DEGs that passed our filtering criteria were detected (*n* = 184). Only the genes (*n* = 78) detected to be downregulated in Parp14‐deficient mice resulted in GO term identifications. Interestingly, all the identified 12 GO terms were related to inflammation or infection. Key examples of the downregulated genes were the three interferon‐inducible genes (*ifitm1*, *ifitm6*, *ifi27l2a*), two pattern recognition receptor genes (*tlr13*, *clec4n*), one chemokine gene (*cxcl13*), and one cytokine receptor gene (*il7r*). We also detected downregulation of two complement genes (*c2*, *c3*) encoding for the C2 classical pathway component and for the C3 activatory hub of all the complement pathways. At the same time, one alternative pathway gene (*cfb*) encoding for factor B acting upstream of C3 was upregulated in the Parp14‐deficient mice. Abnormal complement system functions could have contributed to the phenotypes observed in Parp14‐deficient mice upon DSS exposure. The complement system has gathered prior interest in the IBD research field,^39^ including experimentation in the DSS model, for example.^40^, ^41^ Taken together, the colon transcriptome of Parp14‐deficient mice had abnormalities in the resting state. In particular, the expression of a number of genes with functional relevance to inflammation and infection responses was hampered. We believe that this reflects the previous published work showcasing Parp14 as a potent transcriptional regulator.^7^, ^8^, ^15^

Parp14 is the subject of an active drug development campaign. Compounds that target the NAD+ binding pocket with subsequent ART activity inhibition, for example,^14^, ^42^ or that target the NAD+ binding pocket with subsequent Parp14 proteolysis (proteolysis targeting chimera)^43^ have been reported. Also, one line of research has focused on compounds that target the Parp14 macrodomains, for example,^44^, ^45^, ^46^ involved in the binding of Parp14 to ADP‐ribose conjugates and removal of them.^5^, ^6^ Therefore, pharmaceutical perturbation of Parp14 functions is being explored at multiple levels, from the overall Parp14 amount to its ART activity and from Parp14‐mediated ADP‐ribose‐dependent molecular scaffolding to the reversal of ADP‐ribosylation‐dependent cell signaling events. First of all, these studies are fueled by the genetic findings that a body‐wide deficiency of Parp14 in mice diminished the intensity of allergic reactions.^11^, ^12^, ^47^ To this end, one ART activity inhibitor was recently shown to reduce pulmonary allergic response in mice^48^ and one ART activity inhibitor entered clinical trials for atopic dermatitis (Phase I, NCT05215808). Secondly, in vitro genetic findings have demonstrated that Parp14 inactivation in macrophages skews them toward a pro‐inflammatory IFN‐γ‐driven M1 phenotype while reducing the IL‐4‐driven M2 phenotype.^13^ Accordingly, one ART activity inhibitor has been explored to reverse the immunosuppressive M2 phenotype of tumor‐associated macrophages.^14^ Also, one recent publication reported on the potency of different Parp14 inhibitors to overcome the chronic IFN‐γ‐associated resistance to anti‐PD‐1 immune checkpoint inhibitor therapy in melanoma.^49^ As of now, no published data are available on Parp14 inhibitors in pre‐clinical models of IBD. Our study with body‐wide Parp14 knockout mice indicates that Parp14 inhibition could be detrimental to IBD. However, we argue that more elaborate experimentation is required, for example, by using the chronic DSS exposure colitis model and possibly other chemical models of IBD, such as the oxazolone model.^17^ Indeed, our genetic experiments tell little of the therapeutic potential of small molecular‐weight compounds to reverse the symptoms of already established pathologies.

In summary, our study in the DSS colitis mouse model of IBD^17^, ^18^ provides compelling evidence that Parp14 has an important role in the maintenance of colon epithelial barrier integrity. The Parp14‐deficient mice had increased sensitivity to DSS colitis. The strong colon epithelial cell expression pattern of Parp14 detected in our study in humans and mice indicates that Parp14 may have functions in non‐immune cells in addition to its important regulatory functions in immune cells.^7^, ^8^, ^9^, ^10^, ^11^, ^12^, ^13^, ^14^, ^15^, ^16^ Our finding of the strong focal histological staining pattern of Parp14 in some IBD patients, together with the recent mRNA level indications of Parp14 as a potential driver gene in UC,^37^, ^38^ highlights the need for bigger cohorts to assess Parp14 as a new prognostic or predictive biomarker in IBD.

## AUTHOR CONTRIBUTIONS

Conceptualization: A.T.P.; Data collection/analysis: M.V., L.P., A.P., R.G.P., M.S., J.L., J.M., H.G., T.R., P.R., D.M.T., and A.T.P.; Supervision: J.L., P.S., P.R., D.M.T., and A.T.P.; Writing – original draft preparation: M.V. and A.T.P.; Writing – review and editing: M.V., L.P., A.P., R.G.P., H.G., P.R., D.M.T., and A.T.P.

## DISCLOSURES

The authors declare no conflicts of interest.

## Supporting information


**Data S1.**.

**Dataset S1.**.

**Dataset S2.**.

**Dataset S3.**.

**Dataset S4.**.

**Dataset S5.**.

## Data Availability

The raw sequencing data from PacBio‐based mouse microbiota profiling have been deposited in the NCBI (https://www.ncbi.nlm.nih.gov) BioProject database with accession number PRJNA977978. The raw bulk mouse tissue RNA‐Seq sequencing data have been deposited in the NCBI (https://www.ncbi.nlm.nih.gov) Gene Expression Omnibus (GEO) database with accession number GSE252812. Bulk human tissue transcriptome data with accession numbers E‐GEOD‐14580 and E‐GEOD‐4183 were accessed using the ArrayExpress database (https://www.ebi.ac.uk/biostudies/arrayexpress). All the other data that support the findings of this study are available in the Materials and Methods, Results, and/or Supplemental Material of this article.
