# Supplementary material for: Body‐wide genetic deficiency of poly(ADP‐ribose) polymerase 14 sensitizes mice to colitis
Source: FASEB J. 2024 Jul 5;38(13):e23775. doi: 10.1096/fj.202400484R (PMC13688048; doi:10.1096/fj.202400484R)
Supplement: Supplementary file 1 [file FSB2-38-e23775-s001.pdf]

SUPPLEMENTARY FIGURES

Body-wide genetic deficiency of poly(ADP-ribose) polymerase 14 sensitizes mice to colitis

Madhukar Vedantham[a], Lauri Polari[b,c,d], Anbu Poosakkannu[a], Rita G. Pinto[a], Moona Sakari[a], Jukka Laine[e], Petra Sipilä[a,d], Jorma Määttä[a,d], Heidi Gerke[a,f,g], Tiia Rissanen[h], Pia Rantakari[a,f,g], Diana M. Toivola[b,c,d], & Arto T. Pulliainen[a*]

[a]Institute of Biomedicine, University of Turku, Turku, Finland

[b]Cell Biology, Biosciences, Faculty of Science and Engineering, Åbo Akademi University, Turku, Finland

[c]InFLAMES Research Flagship Center, Åbo Akademi University, Turku, Finland

[d]Turku Center for Disease Modeling, University of Turku, Turku, Finland

[e]Department of Pathology, Turku University Hospital, Turku, Finland

[f]Turku Bioscience Centre, University of Turku and Åbo Akademi University, Turku, Finland

[g]InFLAMES Research Flagship Center, University of Turku, Turku, Finland

[h]Department of Biostatistics, University of Turku and Turku University Hospital, Finland

*Corresponding author

Arto Tapio Pulliainen, Ph.D.

Institute of Biomedicine, University of Turku, Kiinamyllynkatu 10, FI-20520, Turku, Finland

Phone: +358-40-1586044

E-mail: arto.pulliainen@utu.fi

https://sites.utu.fi/turkucellularmicrobiologylaboratory/

https://orcid.org/0000-0002-9361-8963

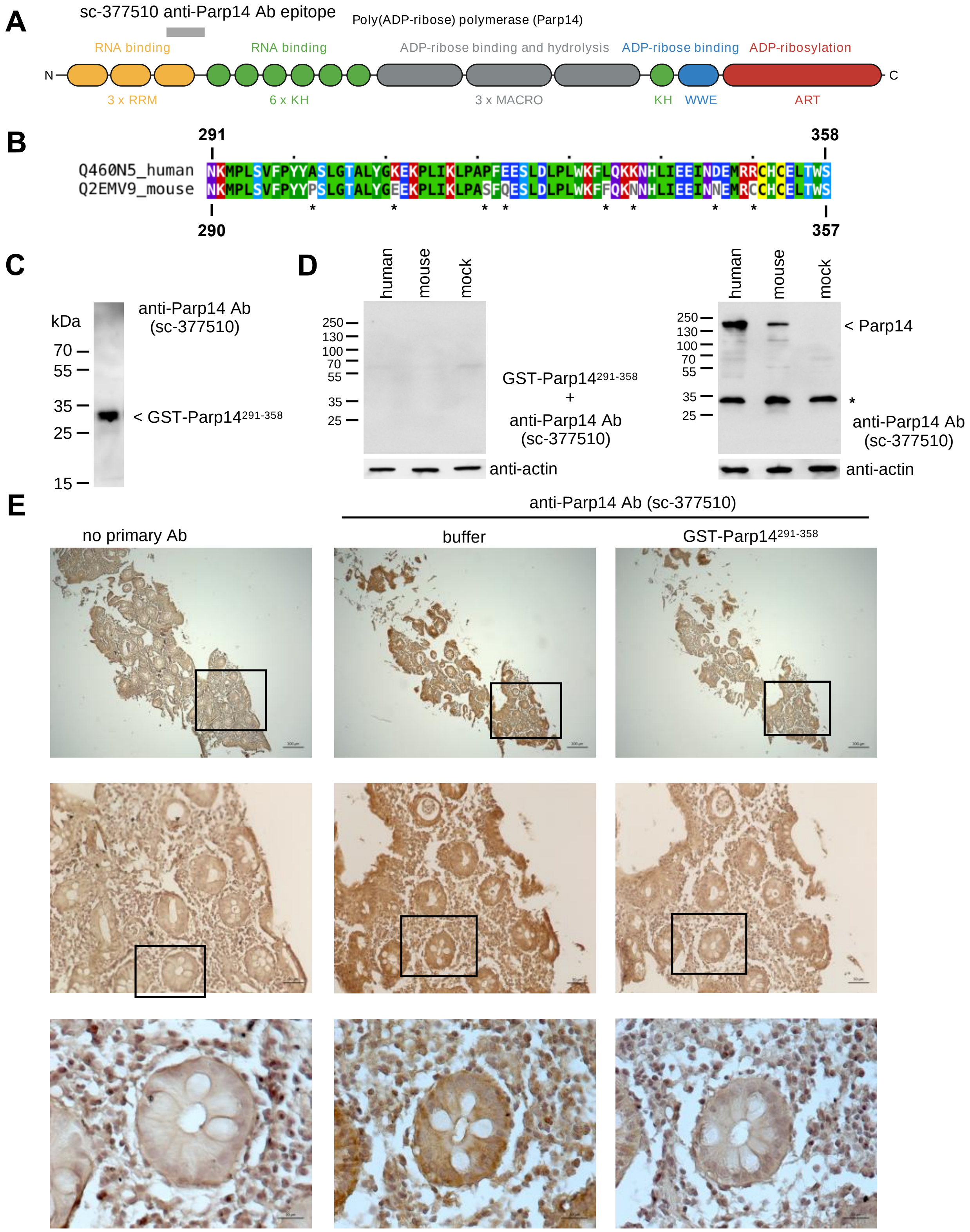

Figure S1. Vedantham et al.

Figure S1. Specificity of the anti-Parp14 antibody. A) The domain structure of Parp14. The localization of the human epitope used to obtain the mouse monoclonal anti-Parp14 antibody (sc-377150, Santa Cruz Biotechnology) is shown with a gray bar. RRM, RNA recognition motif domain; KH, K homology domain; MACRO, macrodomain; WWE, tryptophan-tryptophan-glutamate domain; ART, ADP-ribosyltransferase domain. Image was created with BioRender.com. **B)** Similarities of human and mouse Parp14. This area refers to the epitope of human Parp14 that was used to obtain the mouse monoclonal anti-Parp14 antibody. **C)** Western analysis of the purified GST-Parp14$^{291\text{-}358}$ (human). Expected size of the fusion protein is 34.85 kDa. **D)** Epitope competition Western analysis. Full length human and mouse Parp14 were ectopically expressed in HEK293T cells. The cell lysates were analyzed for Parp14 expression in the presence or absence of GST-Parp14$^{291\text{-}358}$. The star refers to a protein, which consistently stained positive in anti-Parp14 Western analysis of HEK293T cells (see also Fig. S3). **E)** Epitope competition IHC analysis. IHC staining of Parp14 in an FFPE endoscopic biopsy section of an UC patient (TCML_VM_28A, ascending colon, see Suppl. data file 1). 5x, 20x and 40x oil objective images are shown (1:10000 dilution of anti-Parp14 antibody).

no primary Ab

anti-Parp14 Ab (sc-377510)

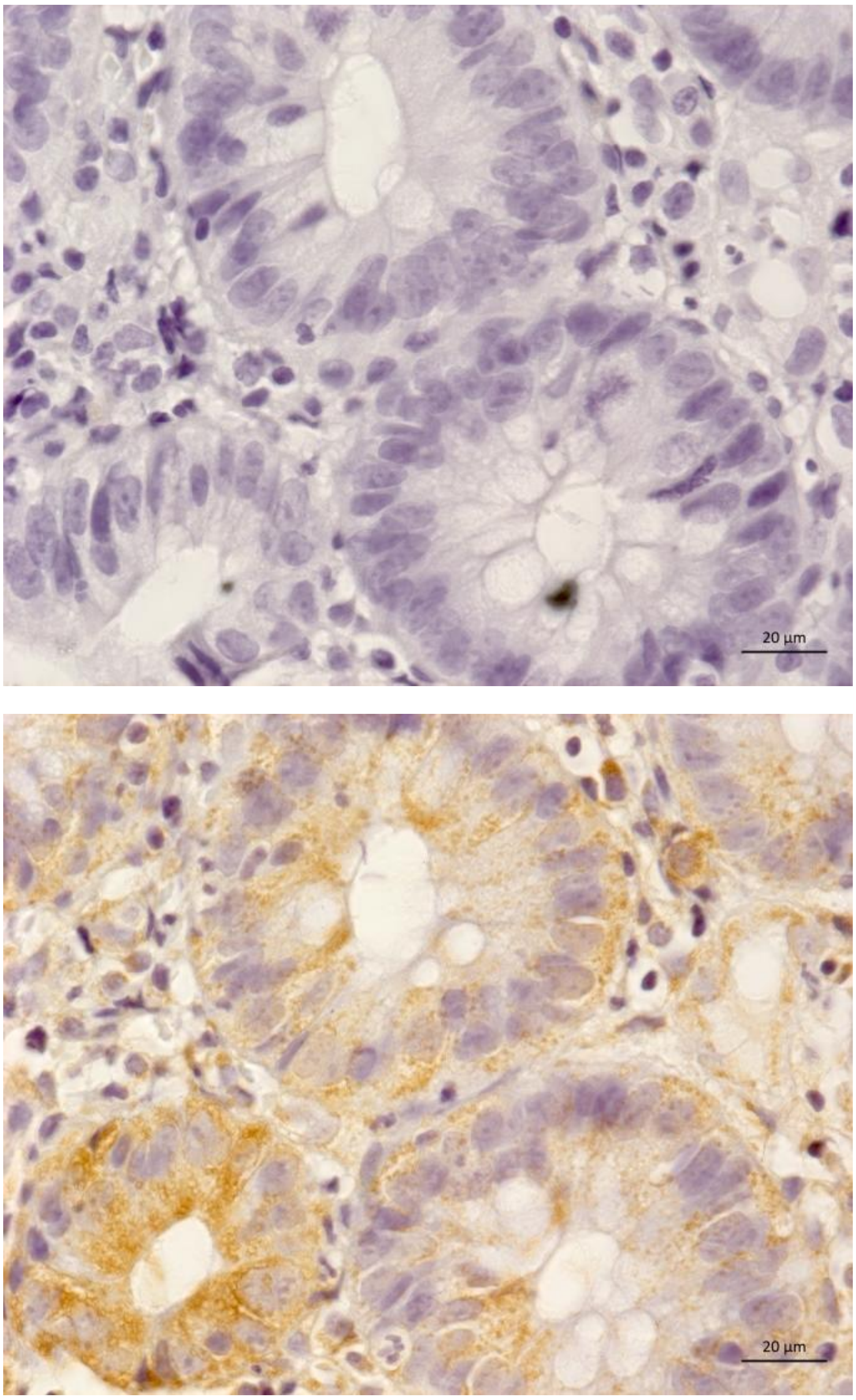

Figure S2. Vedantham et al.

Figure S2. Granular staining pattern of Parp14 in the epithelial cells of human colon. IHC staining of Parp14 in an FFPE endoscopic biopsy section of an UC patient (TCML_VM_21A, ascending colon, see Suppl. data file 1). 63x objective images are shown (1:10000 dilution of anti-Parp14 antibody).

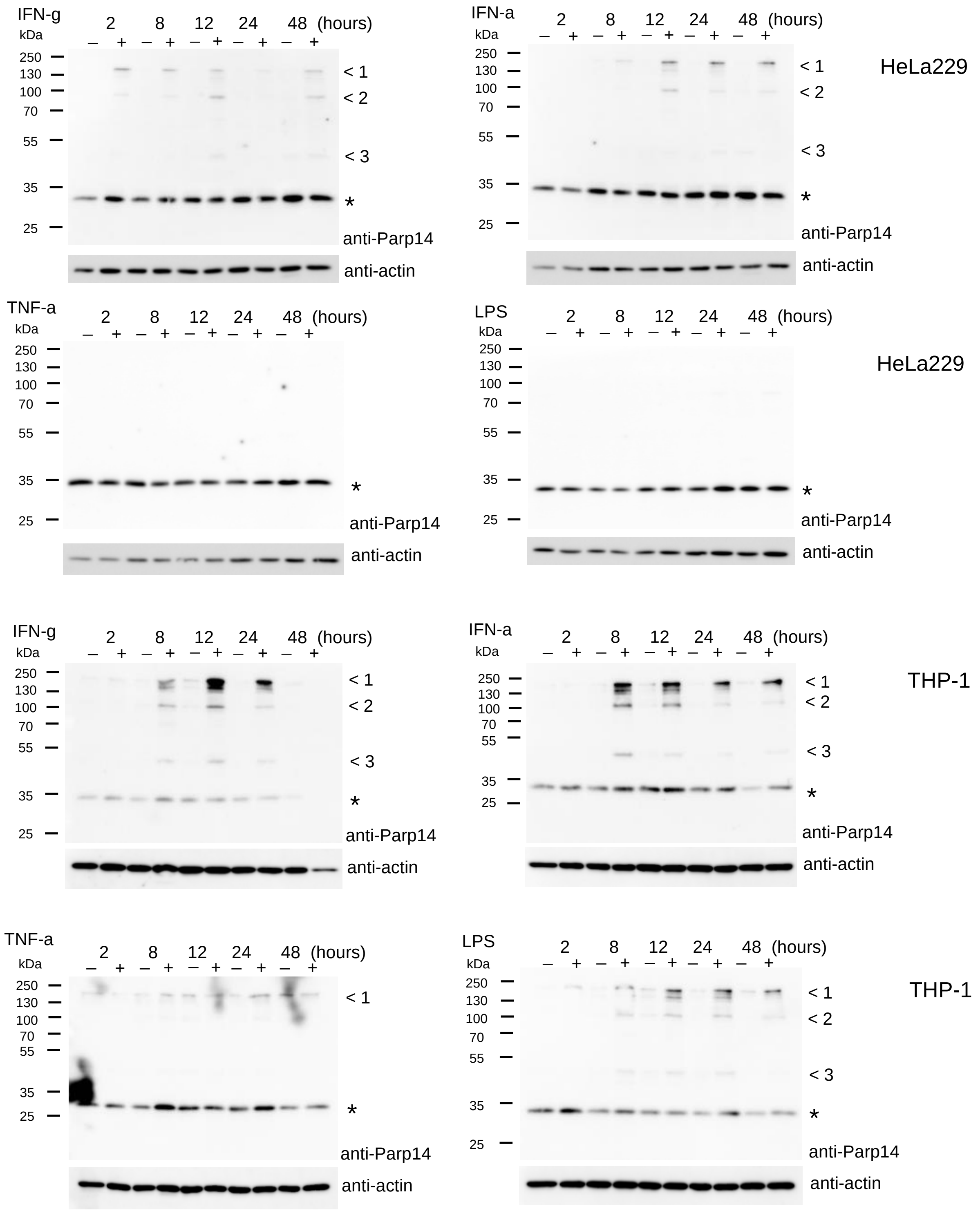

Figure S3. Vedantham et al.

Figure S3. Expression of Parp14 in cultured epithelial cells and macrophages. The cells were either treated with different inflammatory stimuli or left untreated for 2-48 hours (100 U/mL of IFN-γ, 100 U/mL of INF-α, 10 ng/mL of TNF-α or 100 ng/mL of LPS). The cell lysates were analyzed for Parp14 expression using the mouse monoclonal anti-Parp14 antibody. Data with maximum exposure time of 20 min is shown. Loading was controlled with anti-actin Western analysis. Expected size of human Parp14 is 202.83 kDa (Uniprot Q460N5-6, canonical isoform). Arrow 1, full length Parp14; Arrows 2-3, truncated forms or isoforms of Parp14. The star refers to a protein, which consistently stained positive in anti-Parp14 Western analysis with both cell lines.

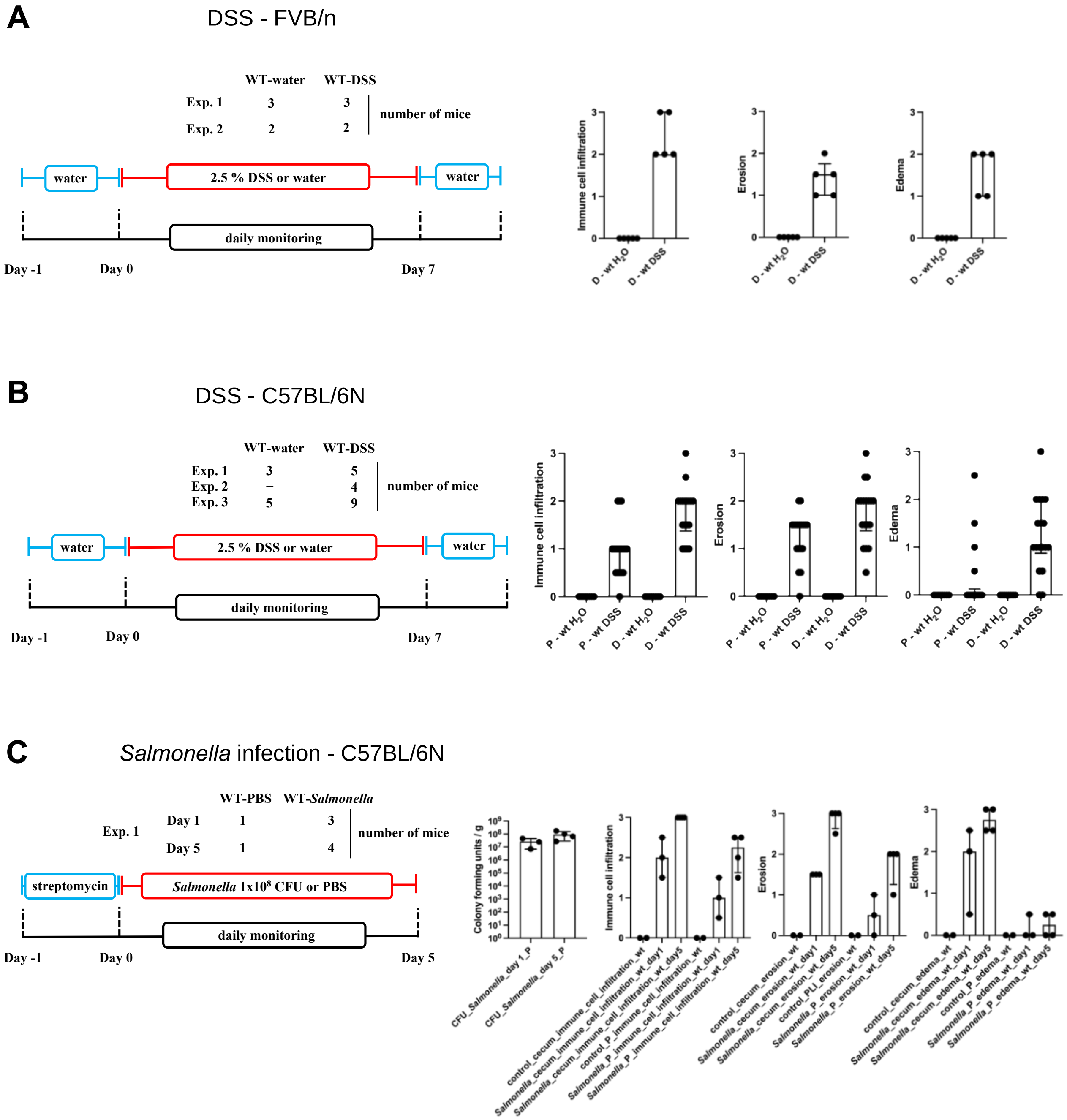

Figure S4. Vedantham et al.

Figure S4. Description of the colitis mouse model experiments. A) DSS exposure in FVB/n background. Two experiments were executed with male wt mice and pooled for the analyses. Quantitation of pathological variables, i.e., immune cell infiltration, epithelial erosion and tissue edema in the distal colon (D), is shown (medians with interquartile range). **B) DSS exposure in C57BL/6N background.** Three different experiments were executed with male mice and pooled for the analyses. Full experimental description with Parp14 deficient mice is shown in Fig. 3A. Quantitation of pathological variables, i.e., immune cell infiltration, epithelial erosion and tissue edema in the proximal (P) and distal (D) colon, is shown (medians with interquartile range). **C) *Salmonella* infection in C57BL/6N background.** One experiment was executed with female mice. This experiment is part of a larger infection experiment with Parp14 deficient mice (Vedantham et al, manuscript in preparation). Quantitation of pathological variables, i.e., number of proximal (P) colon-associated *Salmonella*, immune cell infiltration, epithelial erosion and tissue edema in the cecum (C) and proximal (P) colon, is shown [medians with interquartile range except in colony forming units (CFU) / g graph with mean and standard deviation]. We did not determine the numbers (CFU / g) of cecum(C)-associated *Salmonella*.

no primary Ab

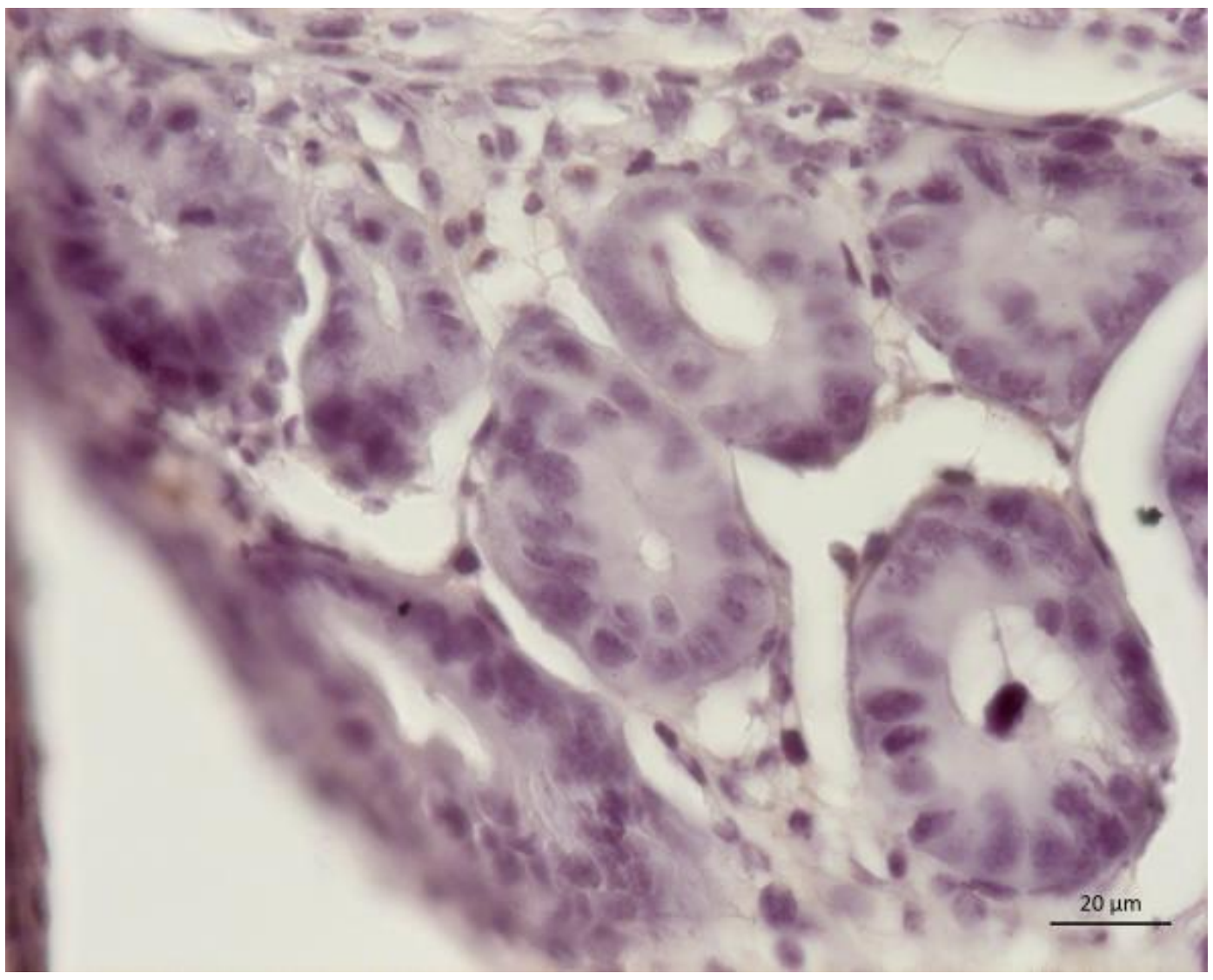

anti-Parp14 Ab (sc-377510)

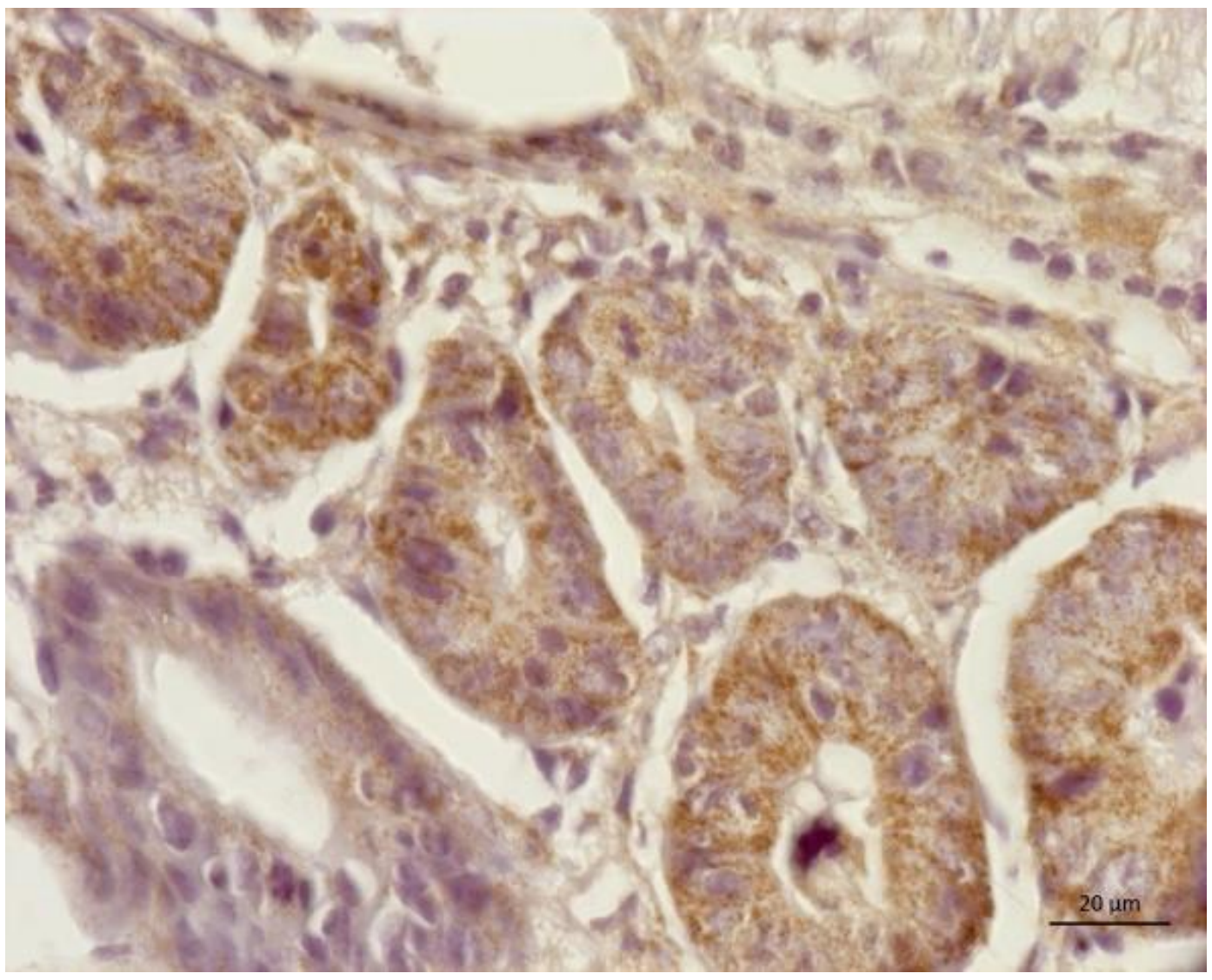

Figure S5. Vedantham et al.

Figure S5. Granular staining pattern of Parp14 in the epithelial cells of mouse distal colon. IHC staining of Parp14 in an FFPE tissue section of a DSS-treated (2.5%, 7 days) FVB/n mice (Fig. S4). 63x objective images are shown (1:500 dilution of anti-Parp14 antibody).

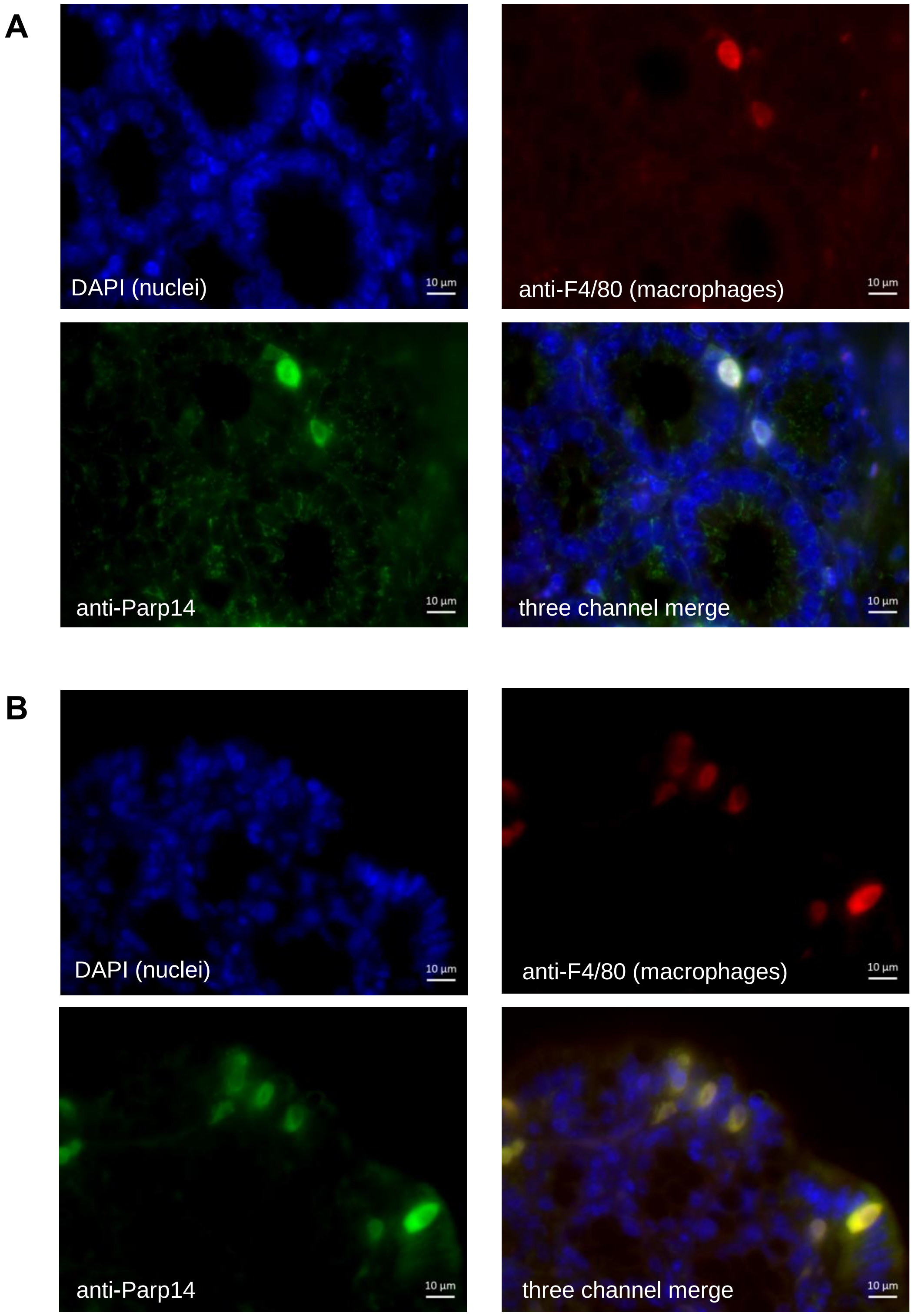

Figure S6. Vedantham et al.

Figure S6. Immunofluorescence imaging of macrophage marker F4/80 and Parp14 in the distal mouse colon. Two different FFPE colon sections (**A-B**) derived from the DSS treated C57BL/6N mice (2.5%, 7 days) were analyzed for Parp14 (green channel) and macrophage marker F4/80 (red channel). 63x objective images are shown (1:500 dilution of anti-Parp14 antibody).

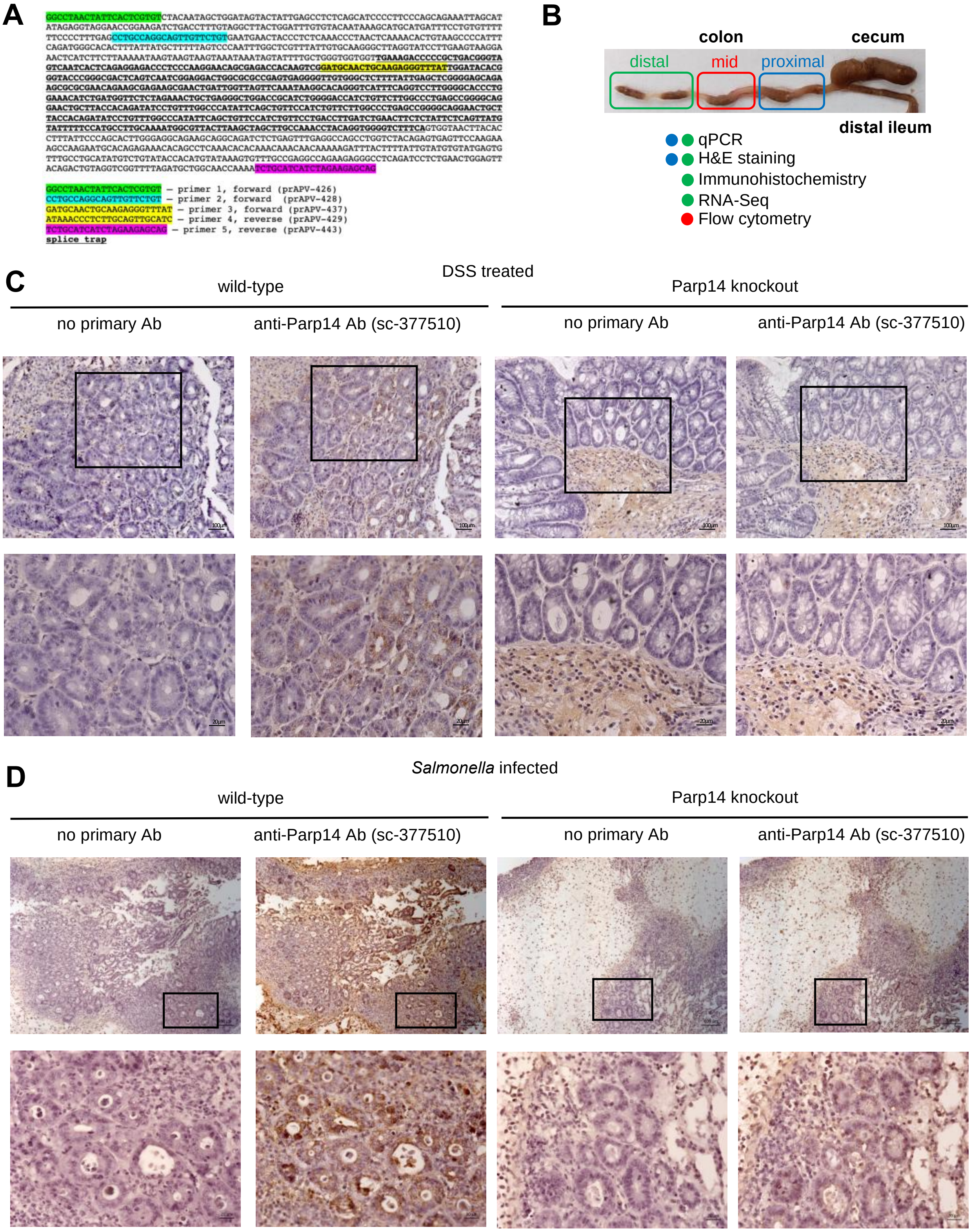

Figure S7. Vedantham et al.

Figure S7. Description of the Parp14 knockout mice, experimental sampling and verification of Parp14 deficiency. A) Nucleotide sequence of the location of the gene trap-targeted mouse *parp14* allele. The gene trap is located between exons 1 and 2. Sequences of the oligonucleotide primers used to genotype the mice during breeding and to sequence the gene trap-targeted allele are shown. **B)** Schematic representation of the sampling for different downstream analysis (see Fig. 3A). **C**) DSS colitis Parp14 IHC data. Parp14 IHC of distal colon of DSS (2.5%, 7 days) treated wt and Parp14-deficient C57BL/6N mice (Exp 3, Fig. 3A). 20x and 40x objective images are shown (1:500 dilution of anti-Parp14 antibody). **D**) Salmonellosis Parp14 IHC data. Parp14 IHC of proximal colon of *Salmonella* infected (5 days) wt and Parp14-deficient C57BL/6N mice (Exp 1, Fig. S4C). 10x and 40x objective images are shown (1:500 dilution of anti-Parp14 antibody).

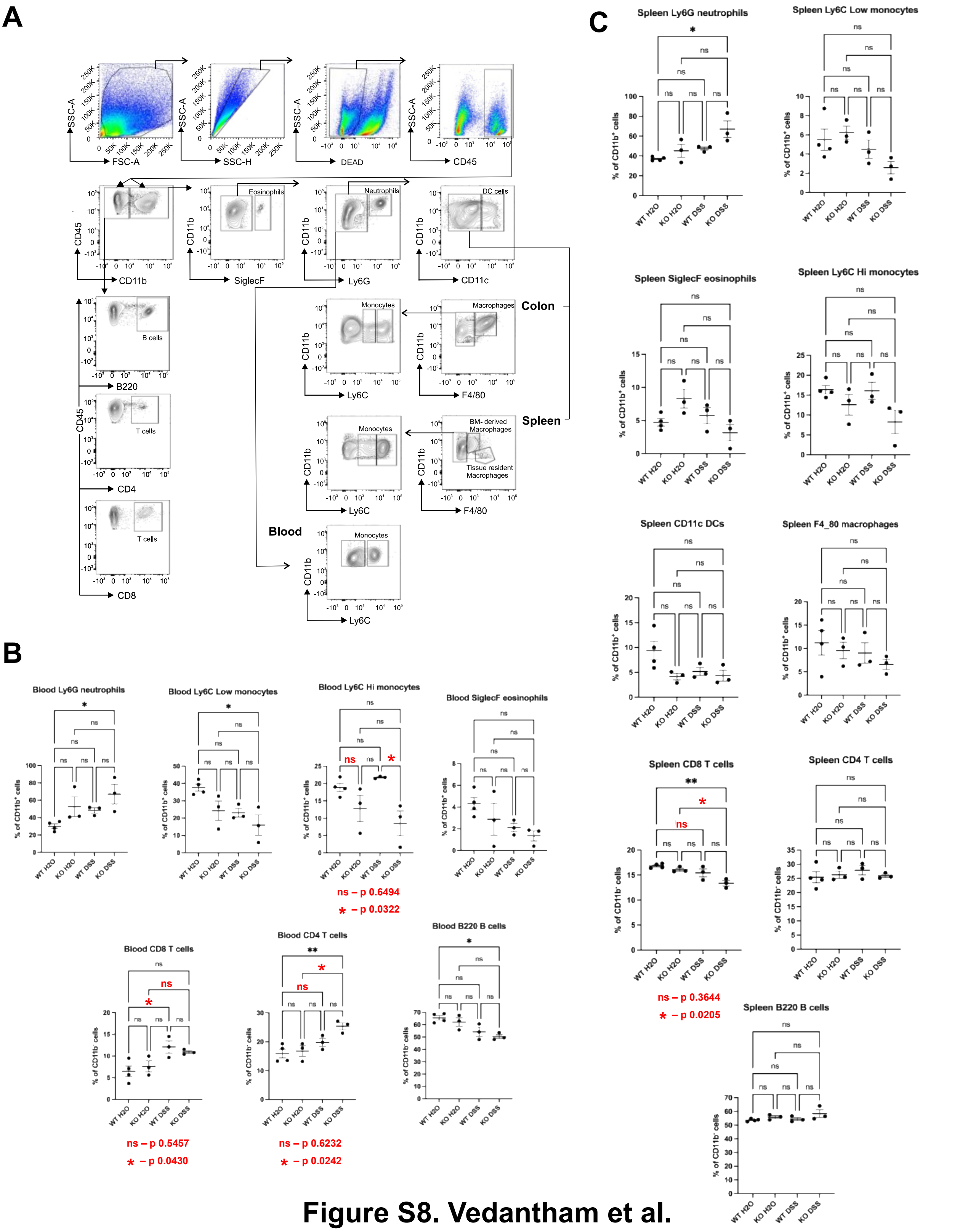

Figure S8. Vedantham et al.

Figure S8. DSS-treated Parp14-deficient mice have abnormal numbers of monocytes and T cells in their blood and spleen. Blood and spleen single-cell suspensions post-DSS and control treatment at day 8 were stained for myeloid-derived cells, i.e., granulocytes (neutrophils and eosinophils), monocytes, macrophages, dendritic cells, and lymphoid-derived cells (B and T cells). **A)** Gating strategy, and results with **B)** blood and **C)** spleen. Each dot represents one mouse from the DSS mouse experiment 3 (Exp 3, Fig. 3A). Data are presented as mean ±SEM. Statistical analysis is done with one-way ANOVA with the Bonferroni post-hoc test (p-values - ns, > 0.05; *, < 0.05; **, < 0.01).

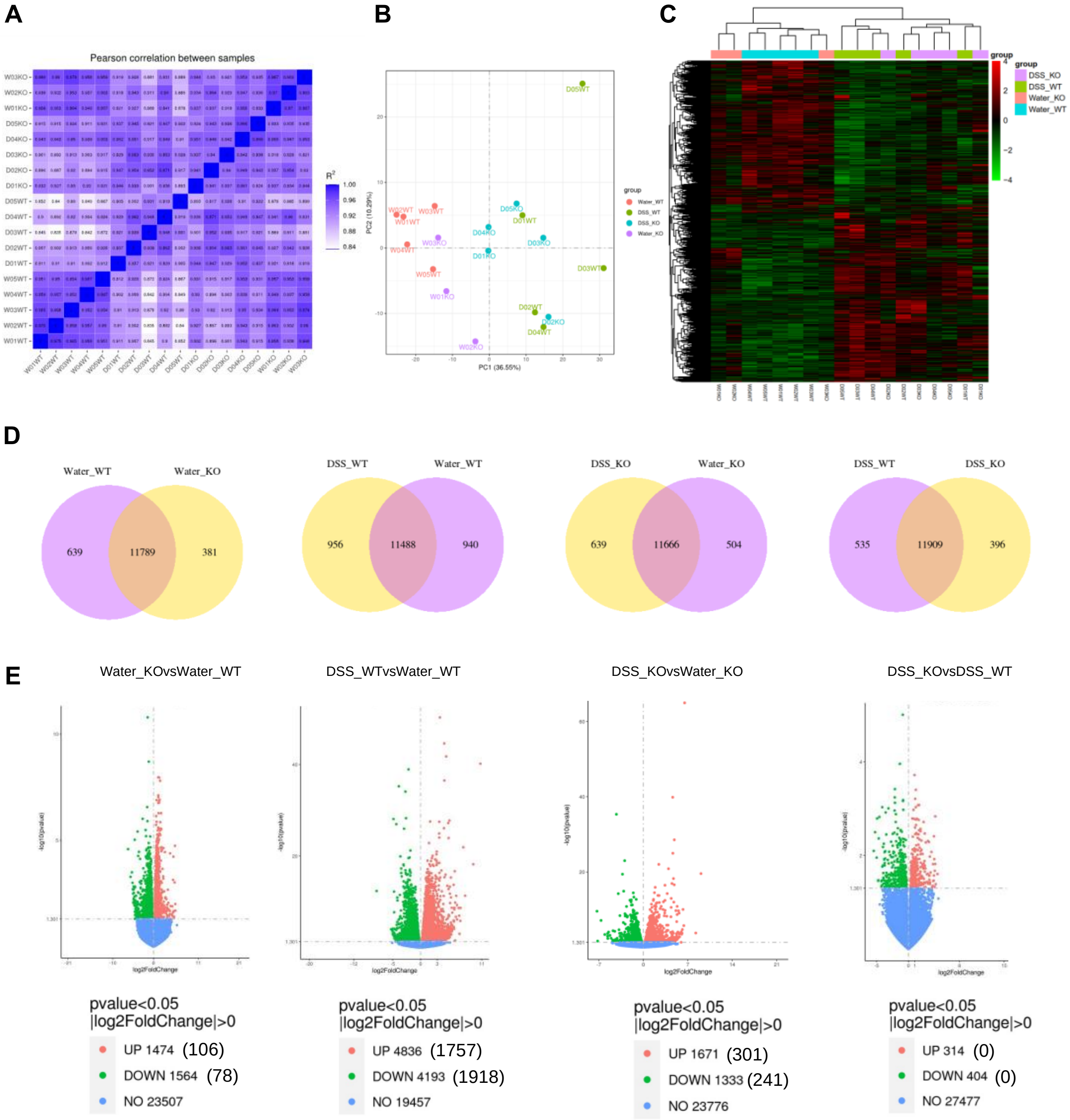

Figure S9. Vedantham et al

Figure S9. The mid-colon bulk tissue RNA-Seq analysis. Data reliability metrics on the different biological replicates (**A-B**) of the DSS mouse experiment 3 (Exp 3, Fig. 3A). Numbers of genes detected to be expressed in the four compare groups, as well as their differential gene expression metrics (**C-E**). **A)** Inter-sample correlation heatmap based on FPKM gene expression values. R^2 is the square of Pearson correlation coefficient (R). R^2 values reflect relatively well the different treatments, e.g., the R^2 values within the water treated wt group are all >0.95 whereas frequently <0.9 when the water treated wt group is compared to the DSS treated wt group. Also, the R^2 values are always >0.9 within the samples of the four compare groups. **B)** Principal component analysis based on FPKM gene expression values. Water- and DSS-treated groups are separated whereas some overlap is visible between the genotypes within both of the treatments. The clear outlier (D05WT) was included into the downstream analysis because of its R^2 values were always >0.9 with the rest of the water treated wt mice (see Fig. S8A). **C)** Heatmap cluster analysis for differential gene expression between the samples. Red color indicates genes with high expression levels, and green color indicates genes with low expression levels. **D)** The Venn diagrams of shared and unique genes that were detected to be expressed among the four compare groups. The integer is the number of shared genes. **E)** Volcano plots of DEGs. Specific information of the DEGs is given in Suppl. data file 3. The x-axis shows the fold difference in gene expression between different samples, and the y-axis shows the statistical significance of the differences. Red dots represent up-regulation genes and green dots represent down-regulation genes. The dashed line indicates the threshold line for statistically significant differential gene expression. The values in brackets refer to the number of DEGs that were used for all the subsequent data analysis after our in-house filtering, i.e., UP genes, log2(FoldChange) > 1 & padj < 0.05; DOWN genes, log2(FoldChange) < -1 & padj < 0.05 (Suppl. data file 2).

A

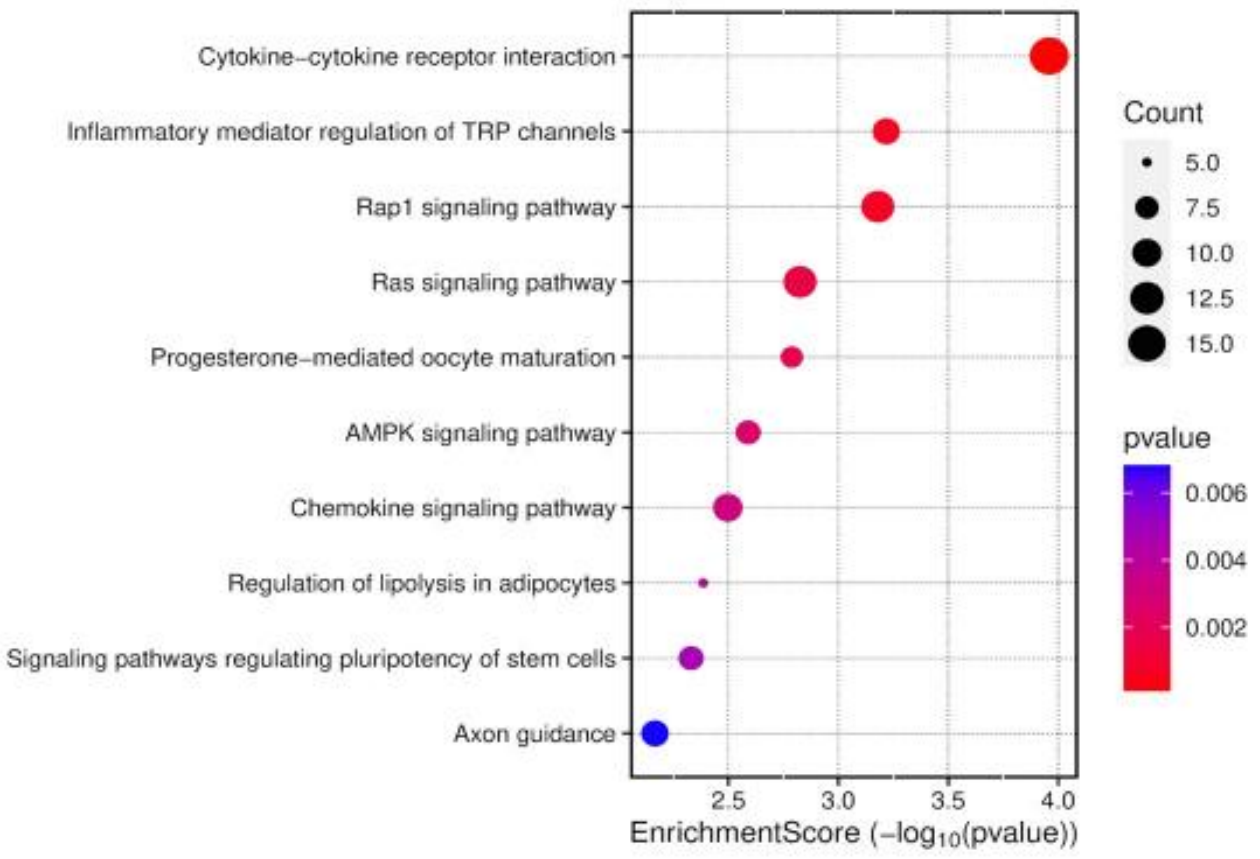

Enrichment dot plot of KEGG pathways

Water_KOvsWater_WT

pathways based on genes detected to be expressed only in the wt mice

B

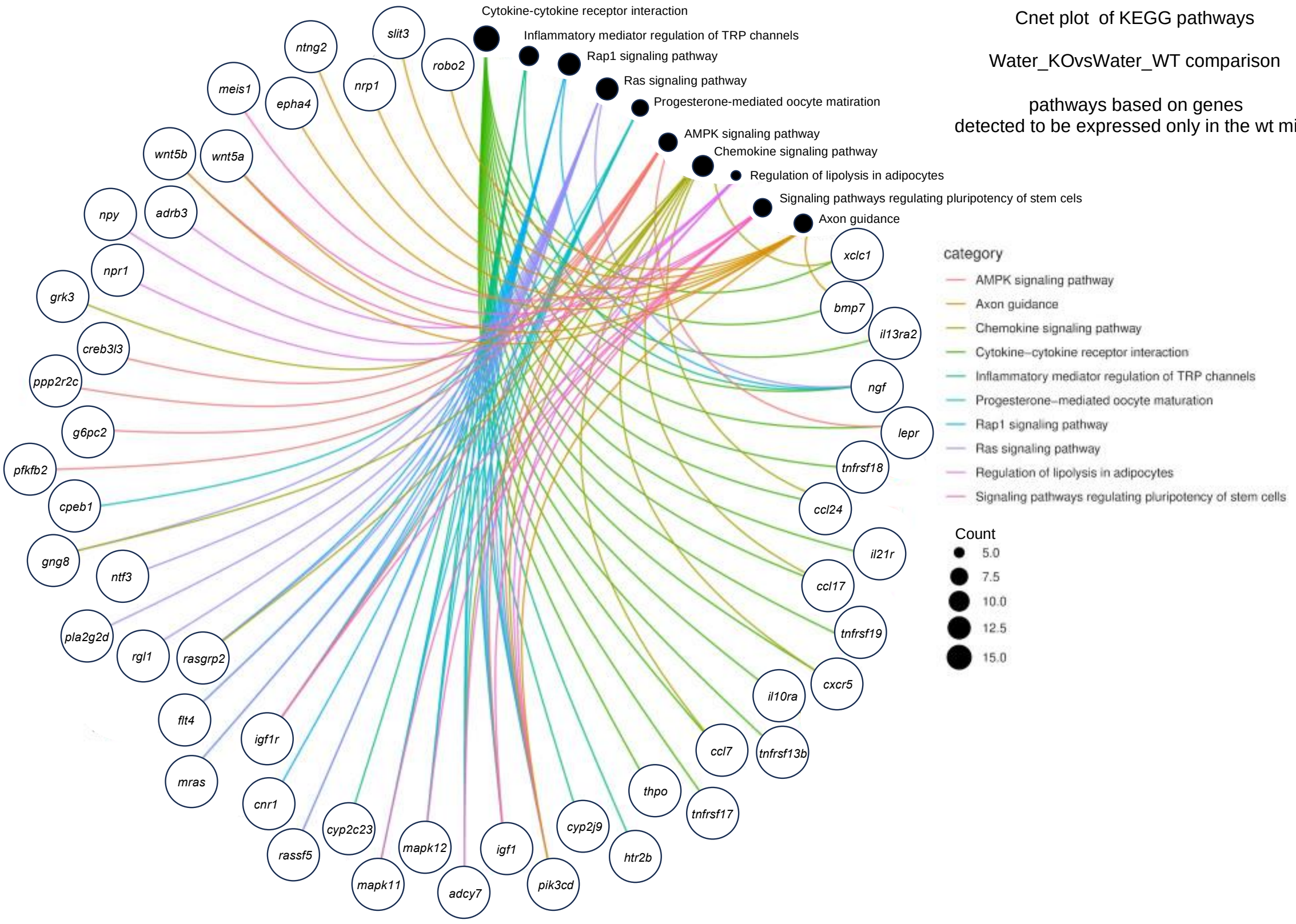

Cnet plot of KEGG pathways

Water_KOvsWater_WT comparison

pathways based on genes detected to be expressed only in the wt mice

Figure S10. Vedantham et al

Figure S10. Parp14-deficient mice have abnormal colon transcriptome in the resting state. Data of a bulk tissue RNA-Seq analysis of the mouse colon distal sections of water-treated wt and Parp14-deficient mice are displayed (DSS mouse experiment 3, Exp 3, Fig. 3A). **A)** Pathway enrichment dot blot representations of the top 10 KEGG pathways identified with genes detected to be expressed only in the wt mice. The KEGG pathways are sorted based on the *p*-value. All the identified KEGG pathways with the corresponding gene lists are described in Suppl. data file 5. The count values refer to the number of genes that were detected in a particular KEGG pathway. **B)** The Cnetplot representation of the top 10 KEGG pathways identified with genes detected to be expressed only in the wt mice. The lines connect the KEGG pathways with the corresponding genes. All the identified KEGG pathways with the corresponding gene lists are described in Suppl. data file 5. The count values refer to the number of genes that were detected in a particular KEGG pathway.

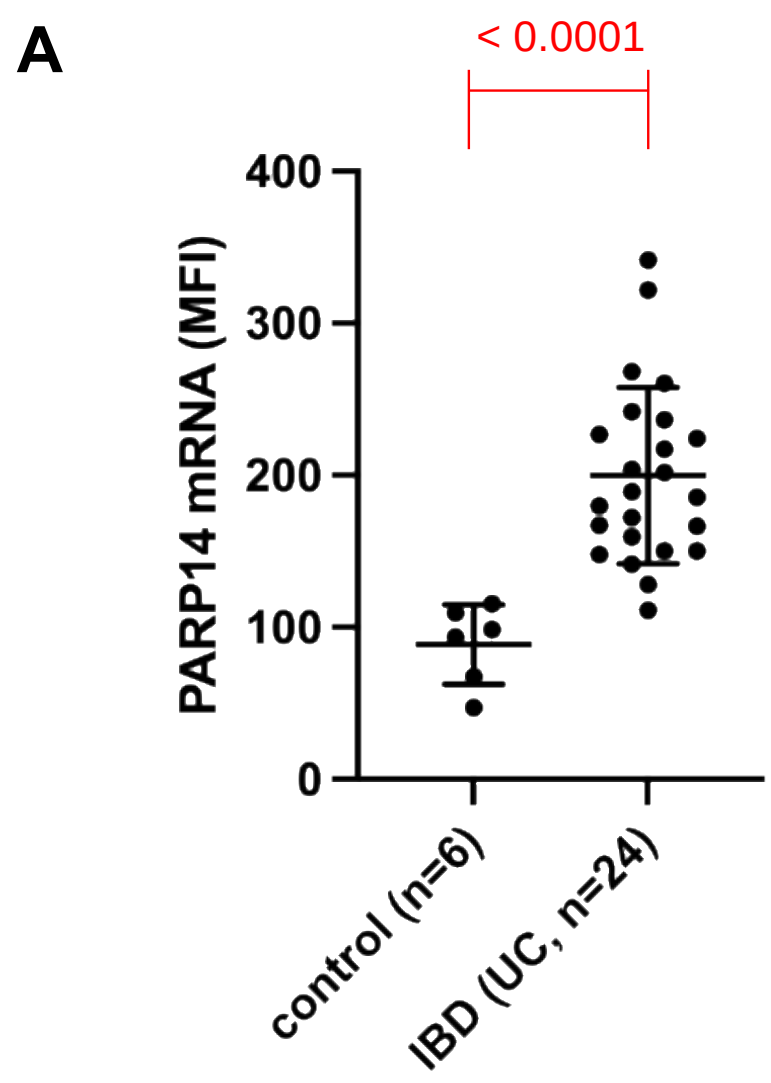

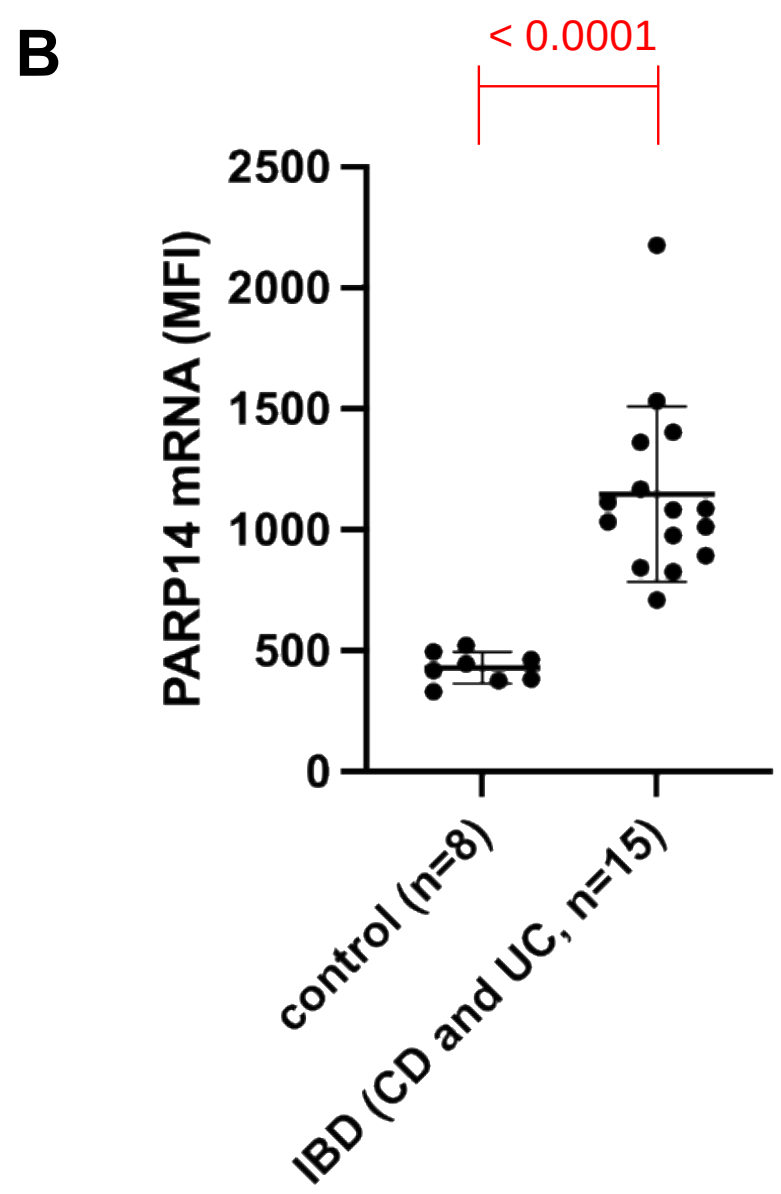

Figure S11. Vedantham et al.

Figure S11. Elevated amount of Parp14 transcripts in IBD patients. Publicly available transcriptome data of colon biopsies from two IBD cohorts are shown for *parp14* transcript levels, i.e., **A)** E-GEOD-14580, and **B)** E-GEOD-4183. Each dot represents a single individual (means with standard deviation, statistics with Mann-Whitney test).
